# Autobiographical memory of validating and invalidating consultations is associated with recall capacity for health information

**DOI:** 10.1371/journal.pone.0353615

**Published:** 2026-07-20

**Authors:** Charlotte E. Lee, Hollie Birkinshaw, Matthew Garner, Tamar Pincus

**Affiliations:** School of Psychology, University of Southampton, Southampton, United Kingdom; University of Bergamo, ITALY

## Abstract

Managing chronic pain involves cognitive and emotional challenges that may affect treatment adherence. Patients’ ability to remember clinician advice is therefore important. Clinician behaviours may influence these processes. To investigate whether recounting autobiographical memories of perceived validating (vs. invalidating) healthcare experiences influences subsequent recall of health-related information in people living with chronic pain, 245 adults with chronic pain (≥3 months) were recruited online. Eligibility criteria included: age ≥ 18 years, fluent in English, no hearing difficulties or mental health conditions, and at least one healthcare visit in the previous two years. Participants were quasi-randomly assigned to describe either a validating or invalidating consultation they had previously experienced. Before and after this task, participants rated their pain intensity and pain-related fear. Participants then listened to 20 health messages and were later asked to recall them in an incidental memory test. The primary outcome was incidental recall of health messages, operationalised as the likelihood of recalling each of the 20 messages. Secondary outcomes included pain intensity and pain-related fear. Recall was analysed using a binomial generalized linear mixed-effects model accounting for individual differences (pain intensity, pain-related fear, immersion in the task, age, sex, and pain duration).Participants who described a validating consultation were more likely to recall health messages than those in the invalidating condition (19% higher odds of recalling each message, based on model estimates). The association was not mediated by change in pain-related fear. These findings indicate that remembering validating consultations might boost retention of health information, highlighting clinician-validation as a potential strategy to support cognitive functioning in people living with chronic pain.

## Introduction

Living with pain poses challenges at individual, interpersonal and societal levels. Beyond pain itself, managing a long-term condition places ongoing cognitive and emotional demands on patients. Many people with chronic pain report difficulties with concentration, attention, memory, and executive functions such as planning, organization, thought-control and goal-directed action [1]. These cognitive difficulties can be distressing and may hinder a patient’s ability to manage their condition [2]. Research has shown that patients who struggle to recall treatment, advice and self-management strategies accurately often have poor adherence with medical advice [3,4].

The Narrow Attentional Resources Hypothesis (NARH), also known as the ‘Capacity-Reduction Hypothesis’ or ‘Attentional Capacity Theory’ [5] suggests that memory deficits associated with chronic pain arise because cognitive resources are reallocated toward emotional and ruminative responses to pain, such as catastrophic thinking, leaving fewer resources available for processing other stimuli [5–7]. Other researchers have focused on emotional regulation [8], finding that higher levels of anxiety correlate with poorer memory [2].

Within the broader context of pain-anxiety, pain-related fear describes fearful thoughts and ruminations about the consequences of pain [9]. According to the NARH, when pain-related fear or anxiety are high, patients may allocate additional cognitive resources to monitoring and anticipating pain, further interfering with memory, attention, and executive functioning [5].

Patient’s fear and anxiety are often closely tied to how their concerns are recognised in clinical encounters [10–13]. A review investigating determinants of treatment adherence in Chronic Pain patients identified clinician-patient relationship quality as important [14]. Clinician validation is defined as explicitly conveying to the patient that their pain experience is understood and believed [15]. It has typically been viewed as a non-specific factor within reassurance frameworks, enhancing the effect of therapeutic interventions [16] and a recent review describes it as necessary for reducing pain-related anxiety and fear [15].

To our knowledge, the impact of validation on recall has only been examined in one small experimental study by Carstens et al., [17]. In this study, healthy participants received either validating or invalidating responses from the experimenter during a task involving experimentally induced pain. The validated group demonstrated higher and more accurate recall than the invalidated group. The current study extends this work by examining these effects in individuals living with chronic pain. To closely approximate real-world clinician validation and invalidation, we opted to use participants’ own autobiographical accounts of previous healthcare consultations to reactivate experiences. This reactivation method is widely used in social psychology [18] and is supported by neurological evidence [19]. Importantly, this approach targets perceived validation and invalidation rather than objective features of clinical encounters, as subjective interpretations of interactions shape emotional responses and cognitive interpretations [20,21].

This proof-of-concept study tests the association between perceived validation and invalidation in autobiographical memories of consultations, participants’ recall of health messages, and whether this is mediated by changes in pain-related fear or anxiety. The study has two aims. First, whether recounting a validating consultation compared to an invalidating one, will improve recall of health information. Second, to examine whether this effect is mediated by changes in pain-related fear or anxiety.

## Methods

### Participants

Participants were recruited through Prolific, an online research recruitment platform. Prolific maintains a participant pool in which users undergo verification procedures, including identity and location checks, as well as ongoing data quality monitoring [22]. Participants complete pre-screening questions and provide demographic information which researchers may use to advertise studies to eligible participants. Eligibility criteria for this study required participants to be ≥ 18 years, fluent in English, free from hearing difficulties, have completed more than 20 studies on Prolific, and to have experienced chronic pain for more than three months. In addition, participants needed to have visited a healthcare provider within the last two years and be able to listen to audio through computer speakers or headphones. Those reporting mental health conditions were excluded because Prolific does not differentiate between specific diagnoses. This broad category may therefore include individuals with cognitive decline or impairment, as well as those with clinical anxiety disorders, which could confound recall performance [23]. Data collection took place from 8^th^-30^th^ October 2024. Participants provided written informed consent via checkbox within the study. The study was approved by the University of Southampton Ethics and Research Governance Committee (ERGO: 98387).

To further ensure data quality, two instructional manipulation attention checks were embedded within scales (e.g., “To check you are paying attention, please select ‘always’”) [24]. Within Qualtrics, failed attention checks triggered an automated warning informing participants that two failed checks would result in automatic rejection, in line with Prolific’s Attention Check Policy [25]. Participants who were automatically redirected following this process were treated as withdrawals and were not included in the final dataset. Demographic data were not retrieved from Prolific for these cases.

Submissions were excluded if the entire study was completed implausibly quickly (more than three standard deviations below the mean completion time) or if participants timed out after 77 minutes of inactivity (platform-imposed limit). Participants also completed a retrospective self-report validity check (“*Should we use your data?*”) and rated their ‘immersion’ in the autobiographical memory task. Finally, an open-ended comment box allowed participants to report interruptions or clarify responses. These procedures maximised the likelihood that participants were genuine individuals living with chronic pain, who had experienced validating and/or invalidating consultations, and who engaged fully with the study.

Participants were asked to read an information sheet and then required to give informed consent via a checkbox before taking part. Participants were informed about what would happen during the study, their right to withdraw, the £3.50 reward for completion and the inclusion of attention check questions. Before the main study, participants completed a pre-screening questionnaire to confirm their eligibility based on self-reported pain status (“*You previously responded ‘Yes’ to the question about chronic pain on prolific. For how long have you experienced this pain?”)* and to determine condition assignment. They were asked whether they had experienced validation and/or invalidation from a healthcare provider in the past. Those who reported having experienced both were randomly assigned to a condition via Qualtrics using the question randomisation function. The randomiser was programmed to evenly assign participants to one of the two experimental branches: one where they were asked to recount a validating healthcare experience, or one where they were asked to recount an invalidating healthcare experience. Those who had experienced only one were automatically assigned to the corresponding condition branch. Participants who had not experienced chronic pain for more than 3 months, and/or those who did not report either a validating/invalidating were excluded from the study.

A simulated dataset using the simr package in R [26] was used to estimate the power to detect a small-moderate effect (β = 0.2) in a binomial GLMM with 20 recall items per participant. The simulation suggested that a sample of 200 participants would provide 80% power. To account for potential data loss due to inattentive responses, incomplete submissions, or exclusions during quality checks (e.g., AI-generated text, mismatched descriptions, or outlier responses), we aimed for 300 participants.

### Design and measures

The study employed a mixed design, with one between-subjects variable (Condition: Validation vs. Invalidation) and one within-subjects variable (Timepoint: Baseline [T1] vs. Post-task [T2]).

#### Autobiographical memory task (between-subjects IV).

Depending on assigned condition, participants were asked to write about a previous experience (autobiographical memory) in which they felt a clinician had been validating or invalidating about their pain experiences in an open response text box. Participants were not specifically asked to describe a consultation about their pain, but given the context of the preceding questions (about their pain experience and diagnosis) almost all did. Instructions and examples (presented in Table 1) were designed to be as similar as possible between experimental conditions with limited changes in wording to convey the two concepts (validation and invalidation) to minimise any systematic bias.

**Table 1:** Question text for the Autobiographical memory task in each experimental condition.

Validation	Invalidation
Next, we would like you to tell us about a time in the past where you felt a healthcare provider **validated** your pain experiences. **Pain validation** can be described by the following statements: A healthcare provider made me feel that what I told them **was understandable.** A healthcare provider made me feel that what I told them **made sense**. A healthcare provider made me feel that what I told them **was reasonable.** A healthcare provider **listened to my concerns.** A healthcare provider made me **feel heard.** A healthcare provider made me feel like **I was believed.** For the next few minutes we would like you to describe in as much detail as possible the things that made you feel **validated.** (What did the doctor/nurse/clinician say? What was their body language like? etc.) Please do not include any identifying information about yourself, the hospital/clinic/GP or the healthcare provider.	Next, we would like you to tell us about a time in the past where you felt a healthcare provider **invalidated** your pain experiences. **Pain invalidation** can be described by the following statements: A healthcare provider has made me feel like I **was** **not** **heard.** A healthcare provider has **not listened to my concerns.** A healthcare provider has made me feel like **I was** **not** **believed.** A healthcare provider has made me feel that what I told them **was** **not** **reasonable.** A healthcare provider has made me feel that what I told them **did** **not** **make sense.** A healthcare provider has made me feel that what I told them **was** **not** **understandable.** For the next few minutes we would like you to describe in as much detail as possible the things that made you feel **invalidated.** (What did the doctor/nurse/clinician say? What was their body language like? etc.) Please do not include any identifying information about yourself, the hospital/clinic/GP or the healthcare provider.

#### Health information recall test (DV).

The health information recall test featured a list of 20 messages related to general health (not pain specific) initially collected from National Health Service websites [27,28]. Messages were independently reviewed by two members of the research team who identified systematic variation in their linguistic framing. We therefore categorised messages based on functional intent, distinguishing between ‘action-oriented’ messages, which encouraged health-promoting behaviours using motivational or approach-focussed language (e.g., “*Drink more water*,” “*Go for an eye test*”), and ‘restriction-oriented’ messages, which discouraged unhealthy or risky behaviours using avoidance-focussed language (e.g., “*Avoid junk food*,” “*Limit alcohol intake*”). Where the initial set of messages resulted in disproportionate numbers across categories, messages were adapted or replaced to achieve a balanced distribution between action-oriented and restriction-oriented content (10 per category). We elected to use health messages that reflected real-world general health advice, rather than neutral word stimuli used in previous research [17] to maximise external validity. These decisions were made in discussions with public contributors. Each message was short (*M* = 4.25 words, *SD* = 1.46), easy to understand, and related to common aspects of health and wellness, including nutrition, hygiene, mental wellbeing, and safety. Exercise-related advice was excluded, since treatment plans for some pain conditions include various levels of exercise, and as such, this advice may be more salient for some participants than others. This decision was supported by consultations with public contributors, who highlighted that such advice might unintentionally invalidate persons with pain who do not feel able to exercise regularly, which may in turn bias results. The 20 messages were delivered as audio recordings to best approximate they way health advice may be given in clinical settings. Messages were played in a randomised order with a 500 *ms* pause between messages. Each message was played only once, and the dependent variable (DV) was the likelihood of accurate recall. Instructions for listening to the audio at the encoding phase were “*On the next page you will hear an audio clip about ‘general health tips’. The audio may only be played once, so only click play when you are ready and have your speakers turned up. Please don’t write down or otherwise record any of the information.”*

Instructions for the test phase were *“Now, thinking back to the recording we asked you to listen to earlier, in which some healthcare providers talked about general health tips. Please write down as many of the health tips as you can remember in the boxes below (one per box). More boxes will appear as you fill them in. Don’t worry if you’re not sure about the exact wording or spelling.”*

#### Additional variables.

The Pain Anxiety Symptom Scale short-version (PASS-20; [9] was used to assess pain-related anxiety before and after the autobiographical memory task. Participants responded to 20 statements on a 6-point likert-scale from 0 (*never*) to 5 (*always*). The PASS-20 measures pain-related anxiety symptoms specific to the experience of chronic pain and includes four subscales: Cognitive, Fear, Escape/avoidance and Physiological Anxiety. It has been found to better predict pain, disability, avoidance and complaints than other more general measures of anxiety in chronic pain populations, has good test-retest reliability (α = .75 −.87) and very high correlations with the original subscales (r = .93 −.97) [9].We selected the two most theoretically appropriate subscales to examine as potential mediators. The Fear subscale was chosen as it aligns most closely with the theoretical frameworks outlined in the literature [9]. The scale includes items such as “*When I feel pain I am afraid that something terrible will happen*” and “*I think that if my pain gets too severe it will never decrease*”. It captures anticipatory fear related to pain and its consequences, which is particularly relevant to emotional processing and attentional disruption. The Cognitive subscale was also selected, which captures patients’ perceptions that pain impairs their thinking, including items such as *“I can’t think straight when I am, in pain*” and *“I find it hard to concentrate when I hurt*”.

Potential confounding variables included pain intensity ratings, measured using the Numeric Pain Rating Scale (NPRS) [29] where participants rated their current, best, and worst pain over the past 24 hours on an 11-point scale from 0 (*no pain*) to 10 (*worst pain imaginable*); the NPRS demonstrates good test-retest reliability for both literate and illiterate individuals with chronic pain (*r* = .95 and.96, respectively) [30]. Diagnostic uncertainty was assessed with questions about participants’ beliefs regarding their diagnosis and explanation, such as “*Do you believe a clear diagnosis was given?*” and “*Do you agree with the explanation provided?*” [31]. Immersion in the autobiographical memory task was measured with a single-item question: “*how much did you feel like you were reliving the experience that you described? (e.g., to what extent did you feel the same feelings that you had in that moment?)*” rated from 0% (*not at all*) to 100% (*completely*). Although this item has not been formally validated, it was included as a theoretically grounded proxy for the subjective experience of reliving the memory (i.e., immersion). Participant age, sex, and pain duration were also recorded as potential confounders.

### Procedure

Participants accessed the study on Qualtrics via a link on Prolific using a laptop, desktop, or mobile device. Functional audio was required, as the recall task was delivered via audio recordings, and participants could also listen to study instructions and descriptions if preferred. This accessibility adaptation was developed based on feedback from public contributors during pilot testing.

Baseline [T1]: Participants first completed a series of baseline assessments including demographic information, details about their pain condition (any diagnoses, duration, affected areas), diagnostic uncertainty, and numeric pain ratings (*current/best/worst* pain levels over the last 24hrs). They also completed the PASS-20 questionnaire. A momentary body awareness questionnaire [32] was administered at this stage as part of a separate study not related to the present research question. This measure assessed self-reported awareness of bodily sensations across seven domains (*heartbeat, breathing, posture, skin, muscles, stomach/intestines, and joints*) on a 0 (*not at all*) to 10 (*very much*) scale. Participants were then offered a short break before proceeding to the next phase of the study.

Experimental Task: Next, participants completed the autobiographical memory writing task, in which they wrote about a past experience of perceived validation or invalidation from a healthcare provider, depending on their assigned condition. Participants were required to write a minimum of 500 characters, and the “*Next*” button appeared only after 3 minutes, ensuring sufficient engagement with the task. However, participants were allowed to spend as much additional time as needed to complete their response.

Post task [T2]: Measures completed at T2 followed immediately after the experimental task within the same study session, with no intervening delay between phases. Participants answered follow-up questions about the healthcare provider and setting they had described and completed the same body awareness questionnaire again (part of the separate study as described above). Participants then listened to 20 audio health messages (identical for all participants) in a randomized order. Randomisation was used to minimise any serial position effects. They subsequently rated their pain and completed the PASS-20 for a second time. Participants then completed two untimed cognitive tasks: a word association task, in which they typed the first word that came to mind in response to neutral or pain-related prompts (part of a separate study and not analysed here), and the incidental recall task, in which they wrote down the health messages they remembered from earlier. At the end of the study, participants rated how immersed they had felt in their past experience during their participation. Participants also were given the opportunity to provide any additional information or comments (optional).

Finally, participants were directed to a debriefing screen which offered information about the study aims, resources for further reading and support links for participants who may have found the reflective writing task emotionally distressing. The study took approximately 30 minutes and participants were paid £3.50 upon completion.

### Patient and public involvement

From the very beginning, this study was shaped by input from a group of public contributors (individuals with lived experience) [33]. Their involvement was essential in defining the study goals and aims, and in highlighting important factors to consider in our analysis, such as the duration of participants’ pain and their current pain levels. They also influenced the study design by advising on clearer wording, font choices to improve readability, and offering audio instructions alongside written ones to make participation easier.

### Statistical approach

To explore whether recounting a validating experience influenced recall accuracy at T2, data were analysed using binomial generalized linear mixed models (GLMMs) implemented in the ‘lme4’ package [34] in R [35]. A binomial GLMM was appropriate because recall was measured for each of the 20 items per participant. Modelling at the item level captures individual differences in memory performance and accounts for the nested structure of items within participants, providing more precise estimates than collapsing responses into a single score per person. Following recommendations by Barr and colleagues [36] the starting model included the ‘maximal’ random effects structure justified by the experimental design to best account for systematic noise. The ‘buildmer’ package [37] was used to identify the best-fitting model. This package ranks predictors by their contribution to model fit and applies a backward elimination procedure, removing variables that do not significantly improve the model. This approach helps to avoid overfitting, retaining only variables associated with meaningful patterns in the data.

## Results

### Data pre-processing

Data collection was initially planned for 300 participants, but was stopped at 288 due to reduced participant availability on Prolific**.** Prior to data processing, participants’ written descriptions of validating and invalidating experiences, as well as their self-reported diagnoses, were carefully checked to ensure relevance and completeness. At this point, 12 descriptions were flagged by researchers as potentially AI-generated due to a high degree of similarity in structure and content. These cases were subsequently reviewed in consultation with the Prolific team following guidance for detecting bots or AI-generated responses [38] and were removed. An additional 3 datasets were removed where the descriptions did not match the prompts given to the participant (e.g., the participant did not describe a validating/invalidating experience or described something other than a visit with a healthcare provider about their pain). Responses to the retrospective self-report validity check were examined, and no participants indicated that their data should not be used; therefore, no additional exclusions were made. Examples of autobiographical descriptions from each group are provided in S1 Appendix.

For each scale, datapoints more than ± 2.5 *SD*s from the mean within each Timepoint × Group were identified as outliers and removed (Pain in the moment 0.73%; Pain across 24 hrs 0.73%; PASS Total 0.37%; PASS Cognitive 2.2%; recall 4.03%), resulting in the exclusion of 28 participants (14 per condition). Data from 245 participants were retained, exceeding the minimum required sample and providing > 80% power to detect small-moderate effects (β = 0.2).

Data were checked for normality, and Cronbach’s alpha indicated good internal consistency for the PASS subscales (Total α = .93; Cognitive α = .88; Escape/Avoidance α = .81; Fear α = .87; Physiological Anxiety α = .82). Diagnostic uncertainty was highly skewed, with 91.02% of participants reporting a diagnosis (Invalidation = 85.95%; Validation = 95.97%), and was therefore not included as a covariate.

### Sample characteristics

During pre-screening, 75.09% of participants reported having experienced both validation and invalidation from a healthcare provider in the past, and were therefore randomly assigned to an experimental condition. The remaining participants reported having experienced only one type of interaction with a healthcare provider (8.42% invalidation only, 16.48% validation only), and were assigned to the corresponding condition automatically.

Participants (*n* = 245; Validation [V] = 124, Invalidation [I] = 121) were aged between 19–76 years (Validation *M* = 41.47, *SD* = 12.00, *Range* = 20–76; Invalidation *M* = 39.40, *SD* = 12.71, *Range* = 19–71). The majority identified as white (70.25%, V = 62.90%, I = 66.53%). Participants lived in 16 different countries, with the majority from United Kingdom (56.73%; V = 57.26%, I = 56.20%), followed by South Africa (17.14%; V = 20.16%, I = 14.05%) and USA (8.98%; V = 8.87%, I = 9.09%). In total they represented 28 distinct nationalities. Sample characteristics are presented in Table 2 and patient-reported characteristics of described healthcare provider are presented in Table 3.

**Table 2:** Sample characteristics.

Category	Subcategory	Invalidation *n* (%)	Validation *n* (%)	Total *n* (%)
Sex	Female	88 (72.7%)	87 (70.2%)	175 (71.4%)
	Male	33 (27.3%)	37 (29.8%)	70 (28.6%)
Pain Duration	3-6 months	4 (3.3%)	5 (4.0%)	9 (3.7%)
	6-12 months	8 (6.6%)	9 (7.3%)	16 (6.5%)
	1-2 years	13 (10.7%)	10 (8.1%)	23 (9.4%)
	2-5 years	27 (22.3%)	29 (23.4%)	56 (22.9%)
	5-10 years	30 (24.8%)	30 (24.2%)	60 (24.5%)
	10-20 years	32 (26.5%)	31 (25.0%)	63 (25.7%)
	More than 20 years	7 (5.8%)	10 (8.1%)	18 (7.4%)
Pain status at baseline	Yes	105 (86.8%)	103 (83.1%)	208 (84.9%)
	No	16 (13.2%)	21 (17.0%)	37 (15.1%)
Pain site	Single site	23 (19.0%)	27 (21.8%)	50 (20.4%)
	Multi-site	98 (81.0%)	97 (78.2%)	195 (79.6%)
Diagnosis status	Yes	104 (86.0%)	119 (96.0%)	223 (91.0%)
	No	17 (14.1%)	5 (4.0%)	22 (9.0%)

**Table 3:** Participant-reported characteristics of described healthcare providers and settings.

Category	Subcategory	Invalidation *n* (%)	Validation *n* (%)	Total *n* (%)
Healthcare Setting	GP Surgery	50 (41.3%)	45 (36.3%)	95 (38.8%)
	Hospital (outpatient)	42 (34.7%)	43 (34.7%)	85 (34.7%)
	Hospital (inpatient)	11 (9.1%)	15 (12.1%)	26 (10.6%)
	Specific pain clinic	5 (4.1%)	9 (7.3%)	14 (5.7%)
	Emergency Department	7 (5.8%)	3 (2.4%)	10 (4.1%)
	Other	6 (4.9%)	9 (7.2%)	15 (6.0%)
HCP Role	Doctor	80 (66.1%)	74 (59.7%)	154 (62.9%)
	Consultant/Specialist	17 (14.0%)	29 (23.4%)	46 (18.8%)
	Nurse	17 (14.0%)	8 (6.5%)	25 (10.2%)
	Other	7 (5.8%)	13 (10.5%)	20 (8.2%)
HCP Age	18-34	27 (22.3%)	25 (20.2%)	52 (21.2%)
	35-44	43 (35.5%)	49 (39.5%)	92 (37.6%)
	45-54	32 (26.4%)	38 (30.6%)	70 (28.6%)
	55+	19 (15.7%)	12 (9.7%)	31 (12.6%)
HCP Gender	Male	65 (53.7%)	72 (58.1%)	137 (55.9%)
	Female	53 (43.8%)	52 (41.9%)	105 (42.9%)
Last Consultation	Within the past year	107 (88.4%)	108 (87.1%)	215 (87.8%)
	1-2 years ago	14 (11.6%)	16 (12.9%)	30 (12.2%)

*Note.* A small number of participants chose not to disclose HCP gender information.

### Paired comparisons

Table 4 presents descriptive statistics for each outcome at baseline (T1) and post-task (T2), separately for the Validation and Invalidation conditions. For each variable, we report the mean and standard deviation at each timepoint, alongside the average change (Δ; calculated as T2 minus T1) within each group. We did not find any significant differences between our experimental groups on any measures at baseline, indicating that randomization was successful.

**Table 4:** Descriptive statistics for measures at Timepoint 1 (baseline) and Timepoint 2 (post-task).

	Validation			Invalidation			Between-group comparison of Δ
Variable	T1 *M* (*SD*)	T2 *M* (*SD*)	Δ	T1 *M* (*SD*)	T2 *M* (*SD*)	Δ	*p*
Pain in the moment (0–10)	4.37 (2.03)	4.44 (2.29)	0.07	4.36 (2.03)	4.12 (2.10)	− 0.24	.151
Average pain across 24 hours (0–10)	4.69 (1.94)	4.72 (2.06)	0.03	4.73 (1.81)	4.56 (1.92)	− 0.17	.151
PASS Total (0–100)	53.52 (19.04)	51.59 (21.83)	− 1.93	53.62 (17.89)	51.92 (19.16)	− 1.70	.774
PASS Cognitive (0–25)	16.77 (5.09)	15.90 (5.84)	− 0.87	17.08 (4.65)	16.69 (5.33)	− 0.39	.288
Pass Escape/Avoidance (0–25)	14.03 (5.39)	13.85 (6.17)	− 0.18	13.96 (5.55)	13.39 (6.01)	− 0.57	.349
PASS Fear (0–25)	12.16 (5.88)	11.46 (7.00)	− 0.70	12.50 (6.27)	12.09 (6.85)	− 0.41	.566
PASS Physiological Anxiety (0–25)	10.56 (6.01)	10.38 (6.35)	− 0.18	10.08 (5.44)	9.75 (5.81)	− 0.33	.666
Immersion (0–100%)		57.80 (29.20)			60.30 (26.90)		.481
Recall Score (0–20)		5.67 (2.05)			5.05 (1.93)		.015 *

*Note.* Independent-samples t-tests were conducted on participant-level change scores to compare differences in change between conditions. *p-*values reflect between-group comparisons of change scores. All p-values were adjusted for multiple comparisons using the Benjamini-Hochberg False Discovery Rate correction (FDR, [39]. Significance (*p* < .05) is denoted by ‘*’.

To evaluate whether the degree of change differed between conditions, we conducted independent-samples *t*-tests on the participant-level change scores (i.e., Δ for each participant) (*p-*values presented in Table 4). Neither group was expected to show notable changes in pain intensity based on our experimental manipulation, findings were consistent with this. For self-reported pain-related anxiety, initial results indicate limited direct effects of the experimental manipulation.

#### Recall.

A rule-based keyword matching algorithm written in R was used to score recall of the 20 predefined health messages. Keywords were derived from the content and intended meaning of each message, and responses were pre-processed (lowercasing and removal of punctuation) before keyword matching using standard string-matching functions. A response was considered correct if it captured the core information or advice from a health message, even if not worded identically. Responses could be credited for multiple distinct messages, but repetitions of the same message were not scored more than once. To validate the scoring procedure, the first author manually scored a random 10% sample of responses (*n* = 162), blind to participant identity and condition. These manual ratings were then compared against the automated scoring to assess agreement between human and algorithm-derived coding. Initial discrepancies were used to refine the keyword criteria in the code to better reflect the intended meaning of each message. A second subset (10%) was then re-checked following revisions to the scoring algorithm and a high level of agreement (97.53%) was achieved before full-dataset scoring. Agreement was calculated as simple percentage agreement between manual validation and automated scoring outputs. The full scoring script is available on the Open Science Framework (OSF) [40].

To test whether participants were more likely to recall health messages after describing a validating consultation compared to an invalidating one, we modelled their ability to recall each message (scored as 1 = recalled, 0 = not recalled) using a binomial generalized linear mixed model (GLMM) with a logit link.

Since each participant listened to the same 20 health messages in a randomised order, we included random intercepts for variability due to different participants and for different health messages. We began with a model that included experimental condition, pain ratings and pain-related fear (before and after the task), immersion, age, sex, and duration of pain, along with their interactions with condition. As participants only took part in one condition, we did not include random slopes for condition by participant. This was the maximal random effects structure for our design, including random intercepts for participants and messages to account for their unique influence on recall. The model was then refined using the ‘buildmer’ package [37].

The final model (Table 5) retained two predictors: experimental condition (validation vs. invalidation) and pain duration. The experimental condition and random intercepts were deliberately retained during model simplification to ensure these key factors remained in the analysis. Participants who described a validating consultation had significantly higher odds of recalling health messages compared to those in the invalidating condition (an increase in odds of 18.50%). Pain duration also influenced recall, but the relationship was non-linear: participants who had experienced pain for 2–5 years showed higher odds of recall than those who had experienced pain for shorter or longer periods of time.

**Table 5:** Summary of fixed effects from a binomial GLMM predicting recall of health-related messages.

Fixed effect	β	*SE*	*z*	*p*	*OR* (95% *CI*)	% Change
(Intercept)	−1.24	0.18	−6.71	.000	0.29	
Condition (Validation)	0.17	0.07	2.43	.014*	1.19 (1.03, 1.36)	18.50%
Pain Duration*.L*	0.10	0.07	1.39	.160	1.10 (0.96, 1.27)	10.50%
Pain Duration.*Q*	−0.21	0.07	−2.96	.003 **	0.81 (0.71, 0.93)	−19.0%
Pain Duration.*C*	0.06	0.07	0.83	.433	1.06 (0.93, 1.22)	6.00%

*Note.* OR = Odds Ratio,*.L* = Linear effect,*.Q* = Quadratic effect,*.C* = Cubic effect. Significance is denoted as *p* < .05 (*), *p* < .001 (**).

We repeated this analysis after removing individuals who reported having only experienced one type of consultation (validating or invalidating), resulting in a purely randomised sample (*n* = 186). The model remained significant, and the effect of condition (validation vs invalidation) was slightly stronger than the full dataset – those who were validated were 22% more likely to recall an item.

#### Mediated moderation.

Two mediated moderation models were tested in R using the ‘lavaan’ package [41]. The first model examined whether validation moderated the relationship between baseline pain-related fear and fear following the experimental task, and whether this in turn predicted recall performance. Both models were overidentified with 2 degrees of freedom; therefore, we report the full models in the Supplementary Materials for completeness, but present key parameter estimates here.

There was no evidence of an indirect effect of validation on recall via pain-related fear (β = 0.002, *p* = .781). A significant direct effect of validation on recall was observed (β = 0.62, *p* = .014). The interaction between validation and baseline fear significantly predicted post-task fear (β = 0.10, *p* = .032), indicating a small moderation effect on changes in fear from T1 to T2. The full model is presented in S1 Fig.

The second model examined whether validation moderated the relationship between pain-related cognitive anxiety from T1 to T2, and whether this predicted recall performance. There was no evidence of an indirect effect of validation on recall via cognitive anxiety (β = 0.004, *p* = .736). A significant direct effect of validation on recall was observed (β = 0.61, p = .018). The interaction effect between validation and baseline cognitive anxiety on post-task cognitive anxiety was not significant (β = 0.01, p = .833). The full model is presented in S2 Fig.

These findings suggest that while validation directly influenced recall, this effect was not mediated by changes in pain-related fear or cognitive anxiety.

## Discussion

The present study examined whether recounting autobiographical memories of perceived validation versus invalidation in clinical consultations influenced patients’ ability to recall health messages, and whether this effect was mediated by changes in pain-related fear. Consistent with our predictions, participants who described a validating consultation demonstrated better recall of health information than those who described an invalidating consultation. Contrary to our second prediction, the effect of validation on recall was not mediated by changes in pain-related fear. Although validation was associated with a small moderating increase in fear from baseline to post-task, this effect did not predict recall performance. Overall, these results suggest that patients’ feelings of validation in clinical settings can have a measurable impact on their ability to retain health-related information, and highlight the potential for using autobiographical memory of consultations as an experimental methodology in people living with chronic pain, providing a safe and ethically sound approach to investigate these effects.

Specifically, writing about an autobiographical memory of a previous validating clinical consultation, compared to an invalidating one, was associated with an approximately 19% increase in the odds of recalling health-related messages in people living with chronic pain. This effect remained robust even when restricting the analysis to a purely randomised subset, suggesting that the benefit of validation on memory is reliable. The recall advantage for information presented immediately after recounting a validating consultation may reflect the reactivation of emotions linked to those clinical encounters [18]. As such, this study provides proof of concept that patients’ perceived validation and invalidation are linked to memory for health information. To our knowledge, this is the first study to explore this hypothesis.

This study built upon findings from Carstens et al., [17] who recruited healthy students. In line with their results, we found that validation enhanced recall, but our study involved people living with chronic pain and assessed recall of meaningful health messages, further improving ecological validity. Using health messages instead of single words reduced linguistic control but increased ecological validity, better reflecting the types of information patients encounter in real consultations. Similarly, presenting the messages in audio rather than written format increased ecological validity, but may have introduced greater variability in processing compared to text-based presentation. To further investigate whether this translates to advice given within the consultation itself, future research should investigate real or simulated consultations.

Interestingly, we did not find evidence for an indirect mediating pathway through reductions in pain-related fear, which we had identified as a key factor in emotional regulation. Although validation was associated with very small interaction effect on changes in pain-related fear (β = 0.10), this change was in the opposite direction to that expected and did not predict recall performance. Given its small magnitude and lack of association with the primary outcome, this effect is unlikely to represent a meaningful pathway underlying the observed effects on recall.

Based on previous evidence, we would expect to find that cognitive impairment, including memory, is associated with pain catastrophizing [42]. One possible explanation is measurement-related. The PASS fear subscale [9], while widely used, captures an individuals tendency to experience pain-related fear – a trait rather than state measure – and is potentially less sensitive to acute fluctuations in a single test session. To our knowledge, no validated state measure of pain-specific anxiety currently exists. Future research might benefit from developing a state-sensitive instrument tailored to pain-related fear in the moment.

A second explanation is methodological. Our task relied on participants recounting autobiographical memories of past consultations. This indirect memory may evoke weaker effects than a real-time clinical interaction, potentially reducing the impact on pain-related fear in the moment. In this study, we provide evidence that this autobiographical task is sufficient to detect small but statistically significant reductions in pain-related fear related to clinician validation. It is highly likely that during and directly following a consultation where it takes place, clinician validation would have a stronger impact. Future studies could test whether in-situ validation boosts recall and reduces pain-related fear in the moment, and at follow-up.

Whilst we did not find evidence that reductions in either pain-related fear or cognitive anxiety mediated the effect of validation on recall, we recognise that other mechanisms not included in this study may be involved. However, it is also worth considering that validation may have a more direct influence on recall, as suggested by both statistical modelling and the mediation analysis. We suggest that validation may address a fundamental need to communicate pain, which constrains other use of cognitive resources until the individual is certain that they have been heard and understood. This theoretical perspective aligns with the broader evolutionary drive for social support when vulnerable, which underpins the need for validation in situations of distress. This mechanism is related to, but distinct from, existing accounts of perseverance loops driven by worry [6]. Whereas a perseverance loop describes a patient’s preoccupation with explaining or solving their pain, we suggest there is a prior “validation loop,” in which a patient is preoccupied with getting recognition of their pain and its impact.

Communicating a complex and often contested medical need requires patients to repeatedly emphasise their distress to ensure that the urgency of their situation is understood. While treatment may be the eventual goal, the more immediate priority is to relieve the burden of *proving* their suffering. In other words, patients need to feel heard, supported, and accepted in order to move from a position of loneliness in their experience to one of collaboration, where patient and clinician work together toward a shared solution. Once validation is received, motivational and cognitive processes can be reallocated to other tasks, such as recalling health advice. As one of our public contributors noted, “*if you feel invalidated you don’t remember what’s said even if it’s critical for your care*.” Securing validation, therefore, may be a prerequisite for effective care.

Importantly, these two loops are not mutually exclusive and often overlap. A patient may be caught in the validation loop before the perseverance loop emerges, meaning early validation is essential in the pain journey, to interrupt the onset of the perseverance loop. In other cases, the two loops may co-occur, meaning that validation must be achieved alongside a diagnosis, because an explanation of pain offered without a foundation of trust will fail to resolve worry. Consequently, invalidation can exacerbate this dynamic, prolonging validation loop while fuelling the perseverance loop. From this perspective validation should not be seen merely as a non-specific factor, but as an active mechanism with measurable cognitive consequences. While previous research has identified non-specific consultation factors such as reassurance (including listening, building rapport, and providing clear information) as being associated with patient outcomes [43–45], our results suggest that validation in particular may influence cognitive outcomes directly. Patients who feel validated can focus on retaining new information, rather than on ensuring their pain is believed.

### Implications for clinical practice

In practical terms, these findings highlight the potential importance of communication style in supporting patients’ engagement with and retention of health information. The results suggest that validation-related behaviours, such as acknowledging patients’ experiences and conveying understanding [15], may be relevant for how effectively information provided during consultations is processed and later recalled. This may be particularly relevant in time-constrained consultations, where patients are required to retain key information after limited exposure. Importantly, the present study examined perceived validation rather than directly observed clinician behaviours, therefore future research is needed to examine the effects of these behaviours directly.

### Methodological limitations

Some further limitations of the current methodology should be noted. First, in the present study, we deliberately excluded participants with diagnosed mental health conditions. Consequently, those most likely to exhibit indirect effects through fear or worry may have been underrepresented. This sampling characteristic offers a plausible explanation for why we did not observe the indirect pathway via reductions in pain-related fear, and further supports the notion that validation itself can have a direct and functionally significant impact on cognitive processes such as recall.

Second is the decision to conduct this proof-of-concept study online. While online samples are more difficult to verify in terms of authenticity, several steps were taken to ensure data quality. Though we cannot be 100% sure that responses in this study were not AI-generated, responses were rigorously checked by the research team, as described in the Methods section. Written descriptions of past experiences included in the final dataset were rich, varied, and consistent with prior qualitative work [43,44]. The platform Prolific is also widely regarded as one of the most reliable options for online research [46,47]. Participants’ chronic pain status and duration were verified through self-report during pre-screening; however, these criteria were not independently confirmed through clinical records or medical assessment.

Our sample has a high proportion of university-educated participants (64.89% held an undergraduate degree or higher), which may not be representative of all people living with pain, particularly as education has been identified as a determinant of patients’ recall of medical advice [23]. Nonetheless, we note that the data collected were consistent with typical pain populations, showing expected correlations (e.g., between pain and pain-anxiety [48]). This approach allowed us to collect a large sample from an international population, which may have included individuals who may have disengaged from traditional healthcare channels. We note that future research should consider potential differences across national contexts and nationalities.

The autobiographical memory task relied on participants’ self-reported descriptions of prior clinical encounters, which reflect subjective and reconstructed perceptions of validation and invalidation rather than objectively verifiable events. As such, it was not possible to independently verify the accuracy or factual content of these accounts. This is a limitation of the present design. However, it is consistent with the theoretical focus of the study, which was concerned specifically with perceived experiences of validation, as these long-term interpretations are likely to shape cognitive and emotional responses in later health contexts. Future research could complement this approach with observational or simulated consultations to further isolate the effects of objectively manipulated validation on memory and recall.

A final limitation to consider is the inclusion of a brief momentary body awareness questionnaire as part of a separate study. Although this measure was not related to the primary cognitive outcomes of this study, it was completed within the same experimental session. Exploratory analyses of this measure, reported in a separate manuscript currently under review, suggest that body awareness was relatively stable over time, with small variations related to experimental condition and levels of immersion in the autobiographical memory task. In the present study, the primary focus was on cognitive outcomes, and this measure was not included as a theoretically driven predictor. Importantly, this questionnaire was administered prior to the encoding of the health messages and under identical conditions for all participants, reducing the likelihood that it differentially influenced experimental groups or the primary outcome of recall.

## Conclusions

In conclusion, the findings from this study suggest that perceived clinician-validation improves recall of health messages compared to clinician-invalidation. These findings may have implications for clinical communication, suggesting that perceived validation during consultations could support patients’ retention of health information. They may also suggest that validation could be an active target mechanism that impacts on cognitive outcomes, rather than merely a non-specific effect. One public contributor emphasised, “*remembering one more thing might not sound like a lot but is really important*”. Given the small magnitude of the observed effects, we recognise that they are unlikely to indicate clinical importance at this stage, and further research exploring the impact of clinical validation in health settings is needed. Future work is required to improve the measurement and manipulation of validation in the lab and in consultations, to explore effects across a range of cognitive, affective and motivational mechanisms [49], and ultimately, to test associations between validation and pain transitions over time.

## Supporting information

S1 Appendix Examples of descriptions given by participants in each experimental condition. (PDF)

S2 Appendix List of members of the Consortium to Research Individual, Interpersonal and Social Influences in Pain (CRIISP). (PDF)

S1 Fig Path diagram of a moderated mediation model examining validation and PASS fear on recall performance. (PDF)

S2 Fig Path diagram of a moderated mediation model examining validation and PASS cognitive on recall performance. (PDF)

## References

[1] Higgins DM, Martin AM, Baker DG, Vasterling JJ, Risbrough V. The relationship between chronic pain and neurocognitive function: a systematic review. Clin J Pain. 2018;34(3):262–75. doi: 10.1097/AJP.0000000000000536 28719507 PMC5771985

[2] Jorge LL, Gerard C, Revel M. Evidences of memory dysfunction and maladaptive coping in chronic low back pain and rheumatoid arthritis patients: challenges for rehabilitation. Eur J Phys Rehabil Med. 2009;45(4):469–77. 20032904

[3] Finestone HM, Yanni MM, Dalzell CJ. Patients’ recall of diagnostic and treatment information improves with use of the Pain Explanation and Treatment Diagram in an outpatient chronic pain clinic. Pain Res Manag. 2015;20(3):145–51. doi: 10.1155/2015/897293 25831077 PMC4447158

[4] Vermeire E, Hearnshaw H, Van Royen P, Denekens J. Patient adherence to treatment: three decades of research. A comprehensive review. J Clin Pharm Ther. 2001;26(5):331–42. doi: 10.1046/j.1365-2710.2001.00363.x 11679023

[5] Eccleston C, Crombez G. Pain demands attention: a cognitive-affective model of the interruptive function of pain. Psychol Bull. 1999;125(3):356–66. doi: 10.1037/0033-2909.125.3.356 10349356

[6] Eccleston C, Crombez G. Worry and chronic pain: a misdirected problem solving model. Pain. 2007;132(3):233–6. doi: 10.1016/j.pain.2007.09.014 17961924

[7] Lefebvre JC, Keefe FJ. Memory for pain: the relationship of pain catastrophizing to the recall of daily rheumatoid arthritis pain. Clin J Pain. 2002;18(1):56–63. doi: 10.1097/00002508-200201000-00009 11803304

[8] Grisart JM, Van der Linden M. Conscious and automatic uses of memory in chronic pain patients. Pain. 2001;94(3):305–13. doi: 10.1016/S0304-3959(01)00366-9 11731067

[9] McCracken LM, Dhingra L. A short version of the Pain Anxiety Symptoms Scale (PASS-20): preliminary development and validity. Pain Res Manag. 2002;7(1):45–50. doi: 10.1155/2002/517163 16231066

[10] Glenton C. Chronic back pain sufferers--striving for the sick role. Soc Sci Med. 2003;57(11):2243–52. doi: 10.1016/s0277-9536(03)00130-8 14512253

[11] Holloway I, Sofaer-Bennett B, Walker J. The stigmatisation of people with chronic back pain. Disabil Rehabil. 2007;29(18):1456–64. doi: 10.1080/09638280601107260 17729093

[12] Walker J, Sofaer B, Holloway I. The experience of chronic back pain: accounts of loss in those seeking help from pain clinics. Eur J Pain. 2006;10(3):199–207. doi: 10.1016/j.ejpain.2005.03.007 16490728

[13] Werner A, Malterud K. It is hard work behaving as a credible patient: encounters between women with chronic pain and their doctors. Soc Sci Med. 2003;57(8):1409–19. doi: 10.1016/s0277-9536(02)00520-8 12927471

[14] Timmerman L, Stronks DL, Groeneweg JG, Huygen FJ. Prevalence and determinants of medication non-adherence in chronic pain patients: a systematic review. Acta Anaesthesiol Scand. 2016;60(4):416–31. doi: 10.1111/aas.12697 26860919

[15] Nicola M, Correia H, Ditchburn G, Drummond PD. Defining pain-validation: the importance of validation in reducing the stresses of chronic pain. Front Pain Res (Lausanne). 2022;3:884335. doi: 10.3389/fpain.2022.884335 36313220 PMC9614309

[16] Vase L, Wager TD, Eccleston C. Opportunities for chronic pain self-management: core psychological principles and neurobiological underpinnings. Lancet. 2025;405(10491):1781–90. doi: 10.1016/S0140-6736(25)00404-0 40382187

[17] Carstens JKP, Boersma K, Schrooten MGS, Linton SJ. Effects of validating communication on recall during a pain-task in healthy participants. Scand J Pain. 2017;17:118–25. doi: 10.1016/j.sjpain.2017.07.003 28850364

[18] Joseph DL, Chan MY, Heintzelman SJ, Tay L, Diener E, Scotney VS. The manipulation of affect: A meta-analysis of affect induction procedures. Psychol Bull. 2020;146(4):355–75. doi: 10.1037/bul0000224 31971408

[19] Suardi A, Sotgiu I, Costa T, Cauda F, Rusconi M. The neural correlates of happiness: a review of PET and fMRI studies using autobiographical recall methods. Cogn Affect Behav Neurosci. 2016;16(3):383–92. doi: 10.3758/s13415-016-0414-7 26912269

[20] Conway MA, Pleydell-Pearce CW. The construction of autobiographical memories in the self-memory system. Psychol Rev. 2000;107(2):261–88. doi: 10.1037/0033-295x.107.2.261 10789197

[21] Lazarus RS, Folkman S. Stress, appraisal, and coping. New York: Springer; 1984.

[22] Prolific. Prolific [Internet]. London, UK: Prolific; First release 2014 [cited 2024 Oct 8]. Available from: https://www.prolific.com

[23] Selic P, Svab I, Repolusk M, Gucek NK. What factors affect patients’ recall of general practitioners’ advice? BMC Fam Pract. 2011;12:141. doi: 10.1186/1471-2296-12-141 22204743 PMC3264522

[24] Oppenheimer DM, Meyvis T, Davidenko N. Instructional manipulation checks: detecting satisficing to increase statistical power. J Exp Soc Psychol. 2009;45:867–72. doi: 10.1016/j.jesp.2009.03.009

[25] Prolific. Attention and Comprehension Check Policy. Prolific [Internet]; 2024 [cited 2024 Jul 10]. Available from: https://researcher-help.prolific.com/en/article/fb63bb

[26] Green P, MacLeod CJ. SIMR: an R package for power analysis of generalized linear mixed models by simulation. Methods Ecol Evol. 2016;7(4):493–8. doi: 10.1111/2041-210x.12504

[27] National Health Service Scotland. Healthy living. NHS Inform [Internet]; 2024 [cited 2024 Jan 18]. Available from: https://www.nhsinform.scot/healthy-living/

[28] National Health Service UK. Live Well. NHS [Internet]; 2024 [cited 2024 Jan 18]. Available from: https://www.nhs.uk/live-well/

[29] McCaffery M, Beebe A. Pain: clinical manual for nursing practice. St. Louis (MO): Mosby; 1989.

[30] Ferraz MB, Quaresma MR, Aquino LR, Atra E, Tugwell P, Goldsmith CH. Reliability of pain scales in the assessment of literate and illiterate patients with rheumatoid arthritis. J Rheumatol. 1990;17(8):1022–4. 2213777

[31] Serbic D, Pincus T. Diagnostic uncertainty and recall bias in chronic low back pain. Pain. 2014;155(8):1540–6. doi: 10.1016/j.pain.2014.04.030 24792624

[32] Walentynowicz M, Erbas Y, Luminet O. Relationship between interoception and emotion regulation in daily life. Psychosom Med. 2022;84(3):A1515. doi: 10.1097/PSY.0000000000001095

[33] Grieve S, Harrison R, Chew-Graham C, Taverner I, Lloyd J, Shivji N, et al. Establishing a public involvement network for chronic pain research in the United Kingdom: lessons learned. Health Expect. 2025;28(4):e70373. doi: 10.1111/hex.70373 40785292 PMC12336369

[34] Bates D, Mächler M, Bolker B, Walker S. Fitting linear mixed-effects models using lme4. J Stat Softw. 2015;67. doi: 10.18637/jss.v067.i01

[35] R Core Team. R: a language and environment for statistical computing. Vienna, Austria: R Core Team; 2024. Available from: https://www.r-project.org/

[36] Barr DJ, Levy R, Scheepers C, Tily HJ. Random effects structure for confirmatory hypothesis testing: keep it maximal. J Mem Lang. 2013;68(3):10.1016/j.jml.2012.11.001. doi: 10.1016/j.jml.2012.11.001 24403724 PMC3881361

[37] Voeten CC. buildmer: Stepwise elimination and term reordering for mixed-effects regression. R package version 2.11; 2023. Available from: https://CRAN.R-project.org/package=buildmer

[38] How prolific detects bots and ai in online research. Prolific [Internet]. [cited 2026 Jun 17]. Available from: https://www.prolific.com/resources/how-prolific-detects-bots-and-ai-in-online-research

[39] Benjamini Y, Hochberg Y. Controlling the false discovery rate: a practical and powerful approach to multiple testing. J R Stat Soc Ser B: Stat Methodol. 1995;57(1):289–300. doi: 10.1111/j.2517-6161.1995.tb02031.x

[40] Lee CE, Birkinshaw H, Garner M, Pincus T. Data repository: clinician validation and recall of health information in chronic pain. Open Science Framework; 2026. Available from: https://osf.io/5ksu9

[41] Rosseel Y. lavaan: An R package for structural equation modeling. J Stat Softw. 2012;48:1–36. doi: 10.18637/jss.v048.i02

[42] Melchior M de O, Antunes LG, Bataglion C, Magri LV. Can high pain intensity and catastrophizing interfere with the cognitive performance of women with chronic pain related TMD? A cross-sectional study. J Appl Oral Sci. 2023;31:e20220384. doi: 10.1590/1678-7757-2022-0384 36995880 PMC10065759

[43] Braeuninger-Weimer K, Anjarwalla N, Pincus T. Discharged and dismissed: a qualitative study with back pain patients discharged without treatment from orthopaedic consultations. Eur J Pain. 2019;23(8):1464–74. doi: 10.1002/ejp.1412 31069890

[44] Holt N, Pincus T, Vogel S. Reassurance during low back pain consultations with GPs: a qualitative study. Br J Gen Pract. 2015;65(639):e692-701. doi: 10.3399/bjgp15X686953 26412846 PMC4582882

[45] Simonsen GD, Jensen TS, Kongsted A. Reassuring patients with low back pain in primary care consultations: does it happen, and does it matter? A ChiCo cohort study. Clin J Pain. 2021;37(8):598–606. doi: 10.1097/AJP.0000000000000946 34010222 PMC8270505

[46] Albert DA, Smilek D. Comparing attentional disengagement between Prolific and MTurk samples. Sci Rep. 2023;13(1):20574. doi: 10.1038/s41598-023-46048-5 37996446 PMC10667324

[47] Douglas BD, Ewell PJ, Brauer M. Data quality in online human-subjects research: Comparisons between MTurk, Prolific, CloudResearch, Qualtrics, and SONA. PLoS ONE. 2023;18:0279720. doi: 10.1371/journal.pone.0279720 PMC10013894 36917576

[48] Rogers AH, Farris SG. A meta-analysis of the associations of elements of the fear-avoidance model of chronic pain with negative affect, depression, anxiety, pain-related disability and pain intensity. Eur J Pain. 2022;26(8):1611–35. doi: 10.1002/ejp.1994 35727200 PMC9541898

[49] Khera T, Rangasamy V. Cognition and pain: a review. Front Psychol. 2021;12:673962. doi: 10.3389/fpsyg.2021.673962 34093370 PMC8175647

