# Supplementary material for: Autobiographical memory of validating and invalidating consultations is associated with recall capacity for health information
Source: PLoS One. 2026 Jul 20;21(7):e0353615. doi: 10.1371/journal.pone.0353615 (PMC13384319; doi:10.1371/journal.pone.0353615)
Supplement: S1 Appendix — (PDF) [file pone.0353615.s001.pdf]

S1 Appendix

Example of descriptions given by participants in the Validation group

"When I was first invited into the GPs room I was immediately put at ease, they spoke softly, offered me a seat and then made direct eye contact as they asked my why I was there (as opposed to other experiences I have had, when the GP fails to make eye contact, speaks harshly and without empathy). The GP listened to everything I had to say without interrupting and then accessed my notes to look at my medical history. They examined my pain with dignity and respect and advised that they could feel exactly where the problem was and had a good idea of what was causing it. They spoke to me like I was human, experiencing the pain, as opposed to simply a problem to be solved and responded accordingly, offering a suggestion of what was happening, possible treatments for both short and long term pain relief and how this might impact me going forward. I left feeling that I wasn't a nuisance and that my symptoms were 'real enough' to warrant the appointment."

Example of descriptions given by participants in the Invalidation group

"The consultant almost dismissed my pain because it is not always there and is very random and occurs all over my body. They gave the impression that it was all in my head and almost dismissed the fact that I was in pain. II also felt they talked down to me as if I didn't understand a thing they were saying. They were very relaxed and gave the impression that they had better things to do. I was given a leaflet and was told to go onto YouTube to look for exercises that they felt might help even though I had already done this research for myself. I was also offered pain management consultations with another department but again I was treated as if I was stupid and have never tried these remedies before. I felt that as soon as a diagnosis had been given that I has just been dismissed"
