# Supplementary material for: Autobiographical memory of validating and invalidating consultations is associated with recall capacity for health information
Source: PLoS One. 2026 Jul 20;21(7):e0353615. doi: 10.1371/journal.pone.0353615 (PMC13384319; doi:10.1371/journal.pone.0353615)
Supplement: S2 Appendix — (PDF) [file pone.0353615.s002.pdf]

Co-Investigators

Professor Ed Keogh

Principle Investigator

Work Packges 1, 5 & 7:

Indivdual, Large cohort & Integration & Translation

University of Bath

Professor Amanda Williams

Co-Investigator

Work Package 6 Lead & 5: Digial & large Cohort

University College London

Professor Candy McCabe

Co-Investigator

Work Package 4: Public Involvement

University of the West of England

Dr Anica Zeyen

Co-investigator

Work Packages 2, 3 & 7: Interpersonal, Societal & Intergration & Translation

Royal Holloway, University of London

Dr Emma Fisher

Co-Investigator

Work Package 1: Indivdual

University of Bath

Dr Tom Fincham-Haines

Co-Investigator

Work Package 7: Intergration & Translation

University of Bath

Professor Chris Eccleston

Co-Investigator

Work Package 7 Lead & 1: Intergration & Translation & Indivdual

University of Bath

Dr Christof Lutteroth

Co-Investigator

Work Package 7: Intergration & Translation

University of Bath

Professor Nadia Berthouze

Co-Investigator

Work Package 6: Digital

University College London

Professor Carolyn Chew-Graham

Co-Investigator

Work Package 4: Public Involvement

Keele University

Professor Rachael Gooberman-Hill

Co-Investigator

Work Package 3 Lead: Societal

University of Bristol

Professor Ernest Choy

Co-Investigator

Work Package 5: Large Cohort

Cardiff University

Professor Rebecca Pearson

Co-Investigator

Work Package 5: Large Cohort

Manchester Metropolitan University

Professor Abbie Jordan

Co-Investigator

Work Package 1, 2 & 7: Indivdual, Interpersonal & Intergration & Translation

University of Bath

Professor Tony Pickering

Co-Investigator

Work Package 7 Lead: Intergration & Translation

University of Bristol

Professor Tamar Pincus

Co-Investigator

Work Package 2 Lead: Interpersonal

University of Southampton

Dr Tom Barry

Co-Investigator

Work Package 5: Large Cohort

University of Bath

Research Fellows & Associates

Dr Hollie Birkinshaw

Research Associate

Work Package 2: Interpersonal

University of Southampton

Dr Diego Vitali

Research Fellow

Work Package 6: Digital Capture

University College London

Dr Amanda Lillywhite

Research Associate

Work Package 7: Integration & Translation

University of Bath

Dr Beate Ehrhardt

Mathematical Innovation Research Fellow

Various Work Packages

University of Bath

Dr Charlotte Lee

Research Fellow

Work Package 2: Interpersonal

University of Southampton

Dr Hannah Sallis

Lecturer

Work Package 3 & 5 Societal & Large Cohorts

University of Bristol

Dr Samantha Stone

Research Associate

Work Package 3: Societal

University of Bristol

Emily Ford

Research Assistant

Work Package 1: Individual

University of Bath

Dr Laura Oporto Lisboa

Mathematical Innovation Research Associate

Work Package 5: Large Cohorts

University of Bath

Dr Sam Hipkiss

Research Associate

Work Package 5: Large Cohorts

University of Bath

Research Collaborators

Dr Jim Dunham

Research Collaborator

Work Package 5 & 7

University of Bristol

Alumni

Dr Emma Begley

Research Associate

Work Package 1: Individual

University of Bath

Dr Victoria Collard

Research Associate

Work Package 5: Large Cohorts

University of Bath

Cara Ghiglieri

Research Associate

Work Package 5: Large Cohorts

University of Aberdeen

Dr Sharon Grieve

Research Fellow

Work Package 4: Public Involvement

University of the West of England

Rosie Harrison

Research Associate

Work Package 4: Public Involvement

Keele University

Dr Amanda Ly

Research Associate

Work Package 3 & 5: Societal & Large Cohorts

University of Bristol

Ellen Readman

PPI Coordinator

Work Package 4: Public Involvement

University of the West of England

Dr Noureen Shivji

Research Associate

Work Package 4: Public Involvement

Keele University

Thompson Tong

Research Assistant

Work Package 7: Integration & Translation

University of Bath

Dr Laura Carter

Research Associate

Work Package 1: Individual

University of Bath

Anna Franklin

Project Coordinator

University of Bath

Anna Gibby

Research Assistant

Work Package 1: Individual

University of Bath

Amber Guest

Research Associate

Work Package 5: Large Cohorts

University of Aberdeen

Adele Higginbottom

PPI Coordinator

Work Package 4: Public Involvement

Keele University

Michelle Robinson

Research Associate

Work Package 4: Public Involvement

Keele University

Juliet Somma

Project Coordinator

University of Bath

Elaine Wainwright

Co-Investigator

Work Package 5: Large Cohorts

University of Aberdeen

Dr Roxanne Cooksey

Research Associate

Work Package 5: Large Cohorts

University of Cardiff

Colin Wilkinson

CPAG Chair

Work Package 4: Public Involvement

Governance Board Members

- Ed Keogh (Bath) (Chair)
- Chris Eccleston (Bath)
- Rachael Gooberman-Hill (Bristol)
- Tony Pickering (Bristol)
- Ernest Choy (Cardiff)
- Carolyn Chew-Graham (Keele)
- Tamar Pincus (Southampton)
- Amanda Williams (UCL)
- Anica Zeyen (RHUL)
- Ian Taverner (CPAG Chair, PPI representative)
- Joanne Lloyd (CPAG Vice-Chair, PPI representative)

Project Management Team Members

Professor Ed Keogh

Principle Investigator

University of Bath

Dr Sarah Eliot

Project Manager

University of Bath
