# Supplementary material for: Autobiographical memory of validating and invalidating consultations is associated with recall capacity for health information
Source: PLoS One. 2026 Jul 20;21(7):e0353615. doi: 10.1371/journal.pone.0353615 (PMC13384319; doi:10.1371/journal.pone.0353615)
Supplement: S1 Fig — (PDF) [file pone.0353615.s003.pdf]

**S2 Fig.** Path Diagram of a Moderated Mediation Model Examining Validation and PASS Fear on Recall Performance.

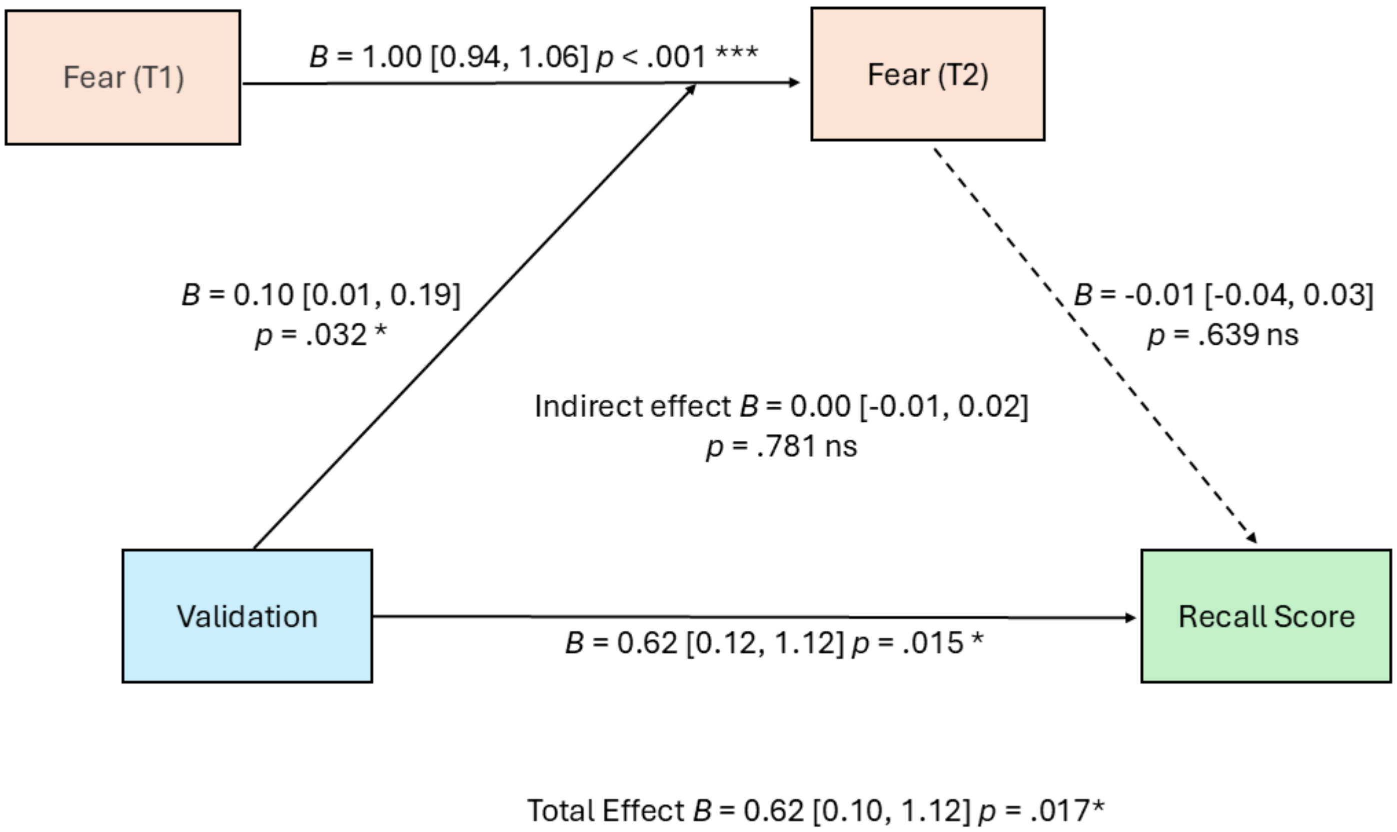


df = 2, CFI = 0.999, RMSEA = 0.036, SRMR = 0.010
