# Supplementary material for: Autobiographical memory of validating and invalidating consultations is associated with recall capacity for health information
Source: PLoS One. 2026 Jul 20;21(7):e0353615. doi: 10.1371/journal.pone.0353615 (PMC13384319; doi:10.1371/journal.pone.0353615)
Supplement: S2 Fig — (PDF) [file pone.0353615.s004.pdf]

S3 Fig. Path Diagram of a Moderated Mediation Model Examining Validation and PASS Cognitive on Recall Performance.

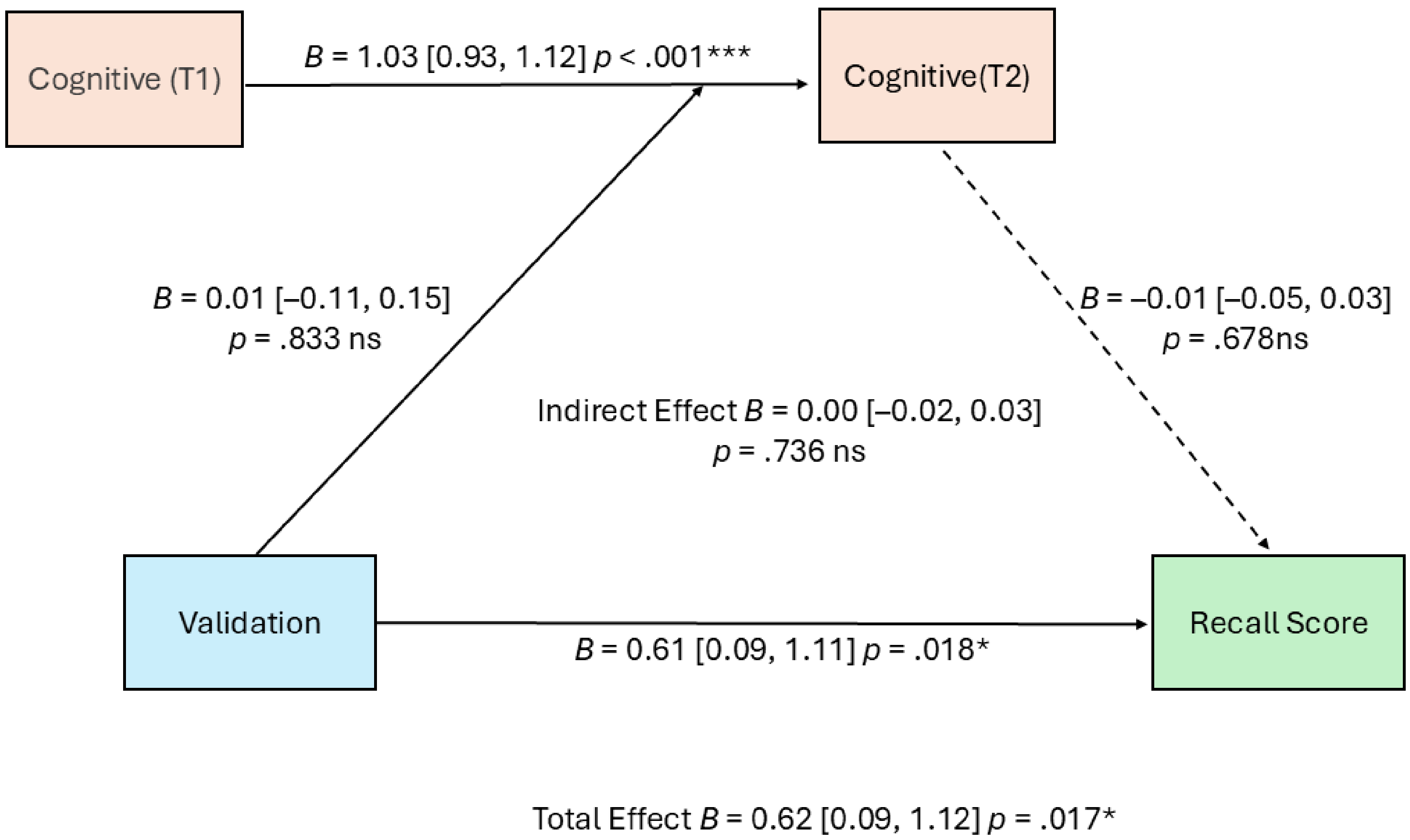

df = 2, CFI = 1.000, RMSEA = 0.000, SRMR = 0.006
