# Supplementary material for: Autobiographical memory of validating and invalidating consultations is associated with recall capacity for health information
Source: PLoS One. 2026 Jul 20;21(7):e0353615. doi: 10.1371/journal.pone.0353615 (PMC13384319; doi:10.1371/journal.pone.0353615)
Supplement: Attachment — Submitted filename: Response to Reviewers.docx [file pone.0353615.s005.docx]

We would like to thank both reviewers for their careful and constructive feedback on our manuscript. We have thoroughly considered all comments and have revised the manuscript accordingly. We believe that these revisions have strengthened the clarity, transparency, and overall quality of the manuscript. Below, we provide a point-by-point response to each comment and indicate where changes have been made in the revised manuscript.

Response to reviewer 1

1. In the abstract, I suggest you to include an opening sentence to contextualize the topic.

We thank the reviewer for this suggestion. We have now added a few sentences to the abstract to give more context (line 17).

2. Line 58, the reference to (Timmerman et al 2016) has to be transformed into a number reference, in line with the others.

Thank you for highlighting this, we have now corrected this reference in line with the journal style.

3. Line 70, I recommend clarifying the connection between what is presented in reference to the literature and the objectives of your study. Written in this way, it is unclear.

We thank the reviewer for this recommendation and have now expanded this section to explain the previous research and how the current study extends these findings in people living with chronic pain (lines 68-79)

4. In general, in the method-participants section, I suggest you to include a specific note explaining in detail how the platform used (Prolific) works, so that even those unfamiliar with it can understand how participants are recruited, how they participate in the research, and how data are collected.

We thank the reviewer for this suggestion and have added some more information on lines 88-93.

*Participants were recruited through Prolific, an online research recruitment platform. Prolific maintains a participant pool in which users undergo verification procedures, including identity and location checks, as well as ongoing data quality monitoring [20]. Participants complete pre-screening questions and provide demographic information which researchers may use to advertise studies to eligible participants.*

5. I suggest you to deeper detail the “payment information” (Line 103).

We have now added “the £3.50 reward for completion” for clarity (line122).

6. In the design and measures section, I suggest you to include the question asked to participants regarding autobiographical memory, so that the task of memory recall is clear to the reader as well. It is not clear if a questionnaire was used, or if there were questions with open response…please clarify this procedure better about the recall task.

We thank the reviewer for this helpful suggestion. We have revised the Design and Measures section to improve clarity regarding the autobiographical memory recall task. Specifically, we have added subheadings for the key tasks and included the full wording of the autobiographical memory instructions for each condition in a new table (Table 1). We have also added the exact instructions for the health information recall task in the Methods section (lines 183-192). These additions clarify that the autobiographical memory task involved open-ended recall rather than questionnaire-based responding.

7. Line 128, you write “To guide this task, examples of validating experiences included:”. Did you check that these examples included in the task did not influence the participants’ description of their autobiographical memories in any way? These examples could have acted as facilitators or suggestions, affecting memory’s dimensions (for example, the vividness of the memory, or the words used to describe the episode) and autobiographical recall. If this check was not carried out, you must include this as a possible limitation of the study.

We thank the reviewer for raising this important point. We acknowledge that the examples provided to illustrate validating and invalidating experiences may have influenced how participants constructed or worded their autobiographical memories. These examples were intended to standardise understanding of the manipulation and were closely matched across conditions to minimise systematic differences in interpretation.

We note that no group differences were observed in immersion ratings (Table 4), suggesting comparable levels of subjective reliving across conditions, although we acknowledge that this does not rule out potential influences of the instructional examples on the content or phrasing of autobiographical memory descriptions.

Importantly, the primary outcome of the present study was recall of separate health information, not the autobiographical memory narratives themselves. Any potential influence of the instructional examples on autobiographical memory descriptions would not be expected to differentially affect the between-group comparison of health information recall, as both groups received structurally equivalent instructions aside from the manipulation.

8. Line 145, you write “These were then adapted to fit two categories:”. Please specify more clearly on what basis this choice was made, in terms of methodology.

We thank the reviewer for this suggestion. We have expanded the explanation of this method of categorisation on lines 161-170. These categories were introduced as part of the message construction procedure based on observed differences in linguistic framing and functional intent of the health messages, and were used solely to ensure systematic development and a balanced stimulus set. They were not included as a factor in the primary analyses and served only to support careful control of message content during stimulus design.

9. Line 186, please specify what you mean by “Immersion in the autobiographical memory task”. Is this question intended to evaluate vividness, accuracy, emotional intensity, or something similar to these characteristics? And in which way can you argue that it is indeed a valid and reliable measure of your construct?
In this paragraph, you talk about “immersion” and then about “emotional engagement”. Please specify what do you mean.

We thank the reviewer for this helpful comment. Immersion in the autobiographical memory task was intended to capture the subjective experience of reliving the autobiographical memory, rather than objective accuracy, narrative detail, or vividness of recall. The wording of the item is provided in the Methods section (lines 129-121)

Although this is a single-item measure and has not been formally psychometrically validated, it was included as a theoretically grounded proxy for reliving during autobiographical recall, consistent with the aims of the study. We have added this clarification on line 221-223.

We agree that the use of “immersion” and “emotional engagement” required clarification. In the manuscript, both terms were intended to refer to the same construct, namely the subjective experience of reliving the autobiographical memory during the task. To improve clarity and consistency, we have standardised terminology throughout the manuscript to “immersion”.

10. Line 202-204 (and Line 215-216), I suggest you to remove “An additional 7-item questionnaire unrelated to the current analysis was also administered at this stage as part of a separate study.” because it is not in line with the study you are presenting in this manuscript.

We thank the reviewer for this suggestion and agree that the reference to the additional 7-item questionnaire is not necessary for the present manuscript give that it took place before the encoding of the recall messages. In line with this recommendation, we have removed this sentence from the Methods section.

11. When you analyzed the memory performance, did you take into account possible serial position effects, such as the primacy effect or the recency effect?

We thank the reviewer for raising this question. The same 20 health messages were presented to all participants. Serial position effects were addressed through randomisation of the presentation order of these messages for each participant. This is now clarified in the manuscript (lines 242-247).

Since items were randomised, any residual serial position effects would be expected to be evenly distributed across groups and are therefore unlikely to have biased the observed between-group comparisons. In addition, item-level differences affecting recall were accounted for in the statistical model by including random intercepts for health messages, ensuring that variability in message difficulty did not influence the estimated effects.

12. Line 263, please specify the “good power” reached by your research.

We have now clarified this on line 301.

13. Please, italicize all letters referring to statistical indicators (e.g., alpha, etc.)

We have now corrected italicisation of statistical indicators in the manuscript for consistency. In line with standard reporting conventions, Greek symbols (e.g., α) have been retained in their standard non-italicised form. However, we are happy to revise if PLoS ONE’s preferred style is for Greek letters to also be italicised.

14. Please specify, the first time you use these letters, that V is for the validation group and I is for the invalidation group.

We have now added this clarification on line 315.

15. Are there differences in distribution by nationality within the groups and in the total sample? How is this monitored, if at all?

We thank the reviewer for this question. Nationality was initially included in the descriptive characteristics of the sample; however, following further consideration and in line with journal data protection guidance, we removed the detailed breakdown due to small subgroup sizes and the risk of potential indirect identifiability when combined with other demographic variables (e.g., age, gender, and education). Aggregated demographic information is now reported in the manuscript in the text on lines 315-321 and in table 2.

Nationality was not included as a covariate in the primary analyses, as more theoretically relevant variables (pain intensity, pain duration, age, and sex) were prioritised. We acknowledge that cultural and national differences may nonetheless be relevant to experiences of validation and invalidation, and we have now noted this as an important direction for future research in the Discussion (line 541).

16. Table 3, p value column is not clear. Does it refer only to the invalidation group? Or to the comparison between the two groups? Do you have p value for the validation group?

Thanks to the reviewer for their attention to this aspect of the table. We have added a clarifying column heading to the table (now Table 4) and included a note specifying that independent-samples t-tests were conducted on participant-level change scores to compare differences in change between conditions. p values reflect between-group comparisons of change scores.

17. Line 304, how was the “custom code written in R” created? What are the methodological foundations and bibliographical references used to create it?

We thank the reviewer for this question and have clarified the development of this scoring code in the manuscript (lines 345-351).

18. Line 309, how many reviewers reviewed the 162 responses? And according to what procedure?

We thank the reviewer for this helpful question. We have revised the manuscript to clarify the scoring validation procedure (lines 351-360). Specifically, the automated scoring was manually validated by a single reviewer (the first author), who was blind to participant identity and condition.

*To validate the scoring procedure, the first author manually scored a random 10 % sample of responses (n = 162), blind to participant identity and condition. These manual ratings were then compared against the automated scoring to assess agreement between human and algorithm-derived coding. Initial discrepancies were used to refine the keyword criteria in the code to better reflect the intended meaning of each message. A second subset (10 %) was then re-checked following revisions to the scoring algorithm and a high level of agreement (97.53 %) was achieved before full-dataset scoring.* Agreement was calculated as simple percentage agreement between manual validation and automated scoring outputs.

19. What type is the indicator of inter-judge agreement regarding the accuracy of revisions made to the random sample?

The indicator reported (97.53%) reflects simple percentage agreement between the manually validated coding and the automated keyword-based scoring procedure. This represents the proportion of cases in which the automated output matched the manual validation within the randomly selected subset of responses. We have clarified this in the revised manuscript (lines 358-359).

20. Line 344, missing an s --> vs

This has now been corrected.

21. The conclusions summarize the results described above. I recommend including sections that focus more on the implications of the study and its connection with the clinical practice.

We thank the reviewer for this helpful suggestion. We have added a section in the Discussion about implications for clinical practice (lines 506-515) and revised the Conclusions section to place greater emphasis on the potential clinical implications of the findings (line 555 – 557). In particular, we now highlight the potential relevance of perceived clinician validation for patients’ recall of health information. We remain cautious about the limited clinical importance of these findings at this stage and emphasise the need for future research in this area.

22. Line 398, I suggest inserting in the text the name of the author you are citing (ref. n. 16).

We have now added the name of the author on line 436.

23. The autobiographical narratives produced by participants in the two groups could be significantly different in terms of length, vividness, quality, accuracy of the memory, the narrative coherence with which they are remembered and narrated, the emotional intensity of each event, the centrality of the specific event for the person, and its social sharing. Were these factors analyzed or taken into account in the analysis and presentation of the results? They may have played a role, even in the subsequent recall of health information.

We thank the reviewer for raising the important point that autobiographical narrative characteristics (e.g., length, vividness, emotional intensity, and coherence) may plausibly influence subsequent memory processes. We agree that these features may contribute to participants’ experiences of the autobiographical recall task, particularly as the narratives differed in content across experimental conditions (validation versus invalidation experiences).

However, the primary aim of the present study was to examine the effect of the experimental manipulation on subsequent outcomes, rather than to model the contribution of individual narrative characteristics. As such, detailed coding and analysis of narrative characteristics was not part of the present analyses and would constitute a separate level of analysis focused on language production rather than memory performance or task-level engagement. Additionally, characteristics such as narrative length or linguistic structure may reflect differences in recall style or expression that are not necessarily equivalent to engagement with the autobiographical experience.

Instead, participants’ self-reported immersion ratings were included as the primary index of engagement during the task, as this measure was designed to directly capture participants’ subjective experience of reliving the autobiographical memory and was considered the most theoretically aligned measure for the aims of this study.

We have clarified in the manuscript that the focus was on the subjective experience of validation/invalidation and its relationship with subsequent outcomes. In addition, we have added a section in the Discussion to address the limitation that autobiographical accounts reflect subjective and reconstructed memories rather than objectively verifiable events (lines 543-552)

24. Line 471, the closing round bracket is missing.

We have now corrected this (Line 539).

Response to Reviewer 2

1. The Introduction section would benefit from further expansion to more clearly situate the study within the existing literature and to better articulate the underlying rationale for the research.

We thank the reviewer for this suggestion and have now expanded the Introduction to include more explanation of previous research and rationale for the current study on lines 68-79)

2. In lines 80–81, the authors state that participants were required “to have experienced chronic pain for more than three months.” Please clarify how the presence and duration of chronic pain were verified, as this appears to rely on self-report.

We thank the reviewer for highlighting this point. The presence and duration of chronic pain were verified through participant self-report during the baseline assessment. As this was an online study, we did not obtain medical records or independent clinical verification of pain diagnoses or duration. We have clarified this in the Methods section (line 124) and added this as a limitation of the study (line 530-533)

3. In lines 90–91, the authors note that “instructional manipulation attention checks were embedded within the study” to ensure data quality. This aspect would benefit from a more detailed description, including examples of the attention checks used and how responses were handled.

We thank the reviewer for this suggestion. We have now expanded the Methods section (lines 104–110) to include an example of the attention checks used and to clarify how failed responses were handled. Specifically, failed checks triggered an automated warning in Qualtrics, and participants were informed that failing two or more attention checks would result in rejection in line with Prolific’s attention check policy. Individuals automatically redirected following failed checks were treated as withdrawals and excluded from analyses.

4. In lines 95–96, the authors indicate that participants completed a retrospective self-report validity check (“Should we use your data?”). Please specify whether any participants responded negatively to this question and, if so, how these cases were treated in the analyses.

We have now added clarification that no participants responded negatively to this question, and as such no additional exclusions were made on lines 293-295.

5. The manuscript does not indicate whether the study was pre-registered. The authors are encouraged to clarify this point and, if the study was not pre-registered, to provide a justification.

We thank the reviewer for this comment. The study was not pre-registered. We note that the study was exploratory in nature, and all materials, analysis code, and data have been made publicly available on the Open Science Framework (OSF) to ensure transparency and reproducibility. A statement has been added to the Data Availability statement (line 575).

6. In lines 158–159, the authors state that “the 20 messages (10 action-oriented, 10 restriction-oriented) were delivered as audio recordings in a randomised order.” Please justify the decision to present the stimuli in audio format rather than in written form, and discuss any potential implications this choice may have for comprehension and recall.

We thank the reviewer for this helpful comment. The decision to present the 20 health messages in audio format was made to enhance ecological validity by approximating how information is typically delivered in clinical consultations. We acknowledge that this may influence encoding and recall, and that audio presentation could differ from written presentation in terms of processing demands. This is therefore noted as a potential limitation (line 441-443). However, we also suggest that it represents a strength of the study in terms of real-world applicability.

7. In lines 202–204, the authors note that an additional 7-item questionnaire, unrelated to the current analysis, was administered as part of a separate study. It would be important to clarify how the authors ensured that these additional items did not interfere with participants’ responses in the present study. Furthermore, for transparency, the content of these items should be reported, either in the main text or as supplementary material.

We thank the reviewer for this comment. The additional 7-item questionnaire assessed momentary body awareness and was administered before the encoding phase as part of a separate study. Iin line with the request from Reviewer 1, we have removed this from the manuscript. As all participants completed the measure under identical conditions, any potential influence would not differentially affect group comparisons.

8. In lines 209–210, the authors report that the “next” button appeared only after 3 minutes. However, earlier (lines 92–93), submissions completed “implausibly quickly” were excluded. The authors should clarify how submissions could be considered implausibly fast given this time restriction.

We thank the reviewer for highlighting this point. We have clarified in the manuscript (lines 111-112) that the exclusion criterion based on “implausibly fast” completions refers to participants who completed the entire study in an unusually short duration (defined as >3 SD below the mean total completion time across the sample). This reflects overall study completion time and is distinct from the task-specific time restriction. The “Next” button delay (minimum 3 minutes) was applied only to the autobiographical recall task to ensure adequate engagement with that component, whereas the exclusion criterion was applied post hoc at the level of total survey duration.

9. The manuscript would benefit from clarification regarding the time interval between T1 and T2. Please specify how much time elapsed between these two measurements.

We thank the reviewer for this comment. The study was conducted within a single continuous online session. Participants first completed baseline measures (T1), followed immediately by the autobiographical recall task, and then proceeded directly to the post-task measures (T2) within the same survey flow. As such, there was no fixed or externally controlled delay between T1 and T2, and the transition between these phases was participant-paced within the Qualtrics platform. For this reason, an exact elapsed interval between T1 and T2 cannot be meaningfully specified as a single value. We have clarified this in the Methods section (lines 242-244)

10. In lines 252–253, the authors state that “12 descriptions were flagged as AI-generated (verified by Prolific) and were removed.” Additional detail is needed on how this verification process operates. Furthermore, please clarify how the authors can be confident that the remaining responses were not AI-generated.

We thank the reviewer for this important point. These responses were initially flagged by the researchers as potentially AI-generated due a high degree of similarity in structure and content. These cases were subsequently reviewed in consultation with the Prolific team following guidance for detecting bots or AI-generated responses <https://www.prolific.com/resources/how-prolific-detects-bots-and-ai-in-online-research>. Based on this the identified responses were removed from the dataset. We have updated the procedure on lines 286-290.

We acknowledge that it is not possible to definitively confirm the origin of all remaining responses. However, Prolific’s participant verification and data quality procedures, combined with our screening and exclusion of suspicious responses, provide reasonable assurance regarding data integrity.

We have added this as a limitation in the Discussion (from line 526-533) and have adopted more cautious wording when describing the written descriptions here.

11. In lines 312–313, the authors report that “a revised subset (10%) was then checked, and a high level of agreement (97.53%) was achieved.” It would be important to specify who conducted this agreement assessment, whether multiple raters were involved, and which metric of agreement was used.

We thank the reviewer for this helpful suggestion. We have revised the manuscript to clarify the scoring validation procedure (lines 351-360). Specifically, that this automated scoring was manually validated by the first author, blind to participant identity and condition.

13. Both Model fit indices indicate excellent fit; however, such perfect or near-perfect fit may raise concerns about model complexity or saturation. The authors should clarify the degrees of freedom and whether the model is just-identified.

We thank the reviewer for this comment. We have now clarified that both models had 2 degrees of freedom and were therefore overidentified rather than just-identified. Although the fit indices indicate excellent model fit, we acknowledge that with such few degrees of freedom, global fit statistics provide limited additional information and should be interpreted with caution. For this reason, we report the full model specifications in the Supplementary Materials and focus primarily on parameter estimates in the main text (lines 392-411). The substantive conclusion remains unchanged: there was no evidence that pain-related fear or cognitive anxiety mediated the effect of validation condition on recall.

12. The finding that validation moderated the change in fear from T1 to T2 warrants further explanation. The authors are encouraged to elaborate on the theoretical and/or empirical rationale underlying this effect.

We thank the reviewer for this helpful comment and for the opportunity to clarify this finding. We did not find evidence that this effect contributed to the indirect pathway to recall performance. We have also corrected an error in the original wording: the effect of validation on changes in pain-related fear was incorrectly described as a small reduction, when in fact it reflected a very small increase. Although validation was associated with a very small interaction effect on changes in pain-related fear (β = 0.10), this effect was in the opposite direction to that expected and did not predict recall. Given its small magnitude and lack of association with the primary outcome, we have clarified in the Discussion that this effect is not theoretically interpreted and should be considered of limited substantive significance (lines 448-452).
