# Supplementary material for: Autobiographical memory of validating and invalidating consultations is associated with recall capacity for health information
Source: PLoS One. 2026 Jul 20;21(7):e0353615. doi: 10.1371/journal.pone.0353615 (PMC13384319; doi:10.1371/journal.pone.0353615)
Supplement: Attachment — Submitted filename: Response_to_reviewers_auresp_2.docx [file pone.0353615.s006.docx]

In accordance with Reviewer 2 (and contrary to Reviewer 1), I invite you to report that an additional 7-item questionnaire was administered as part of a separate study. I agree with Reviewer 2 that this should be done for transparency. I also invite you to briefly describe the content of these items and the main reason why this instrument was not expected to influence your findings.

Thank you for reviewing our revised manuscript, and for your positive comments about the changes made. We agree that transparency is important, and are happy to include more details about the additional questionnaire that formed part of a separate study.

Procedural information was added on lines 234-238 and 250-251:

*A momentary body awareness questionnaire [32] was administered at this stage as part of a separate study not related to the present research question. This measure assessed self-reported awareness of bodily sensations across seven domains (heartbeat, breathing, posture, skin, muscles, stomach/intestines, and joints) on a 0 (not at all) to 10 (very much) scale.*

*Participants answered follow-up questions about the healthcare provider and setting they had described and completed the same body awareness questionnaire again (part of the separate study as described above).*

We have also added a discussion of this as a potential limitation on lines 559-569:

*A final limitation to consider is the inclusion of a brief momentary body awareness questionnaire as part of a separate study. Although this measure was not related to the primary cognitive outcomes of this study, it was completed within the same experimental session. Exploratory analyses of this measure, reported in a separate manuscript currently under review, suggest that body awareness was relatively stable over time, with small variations related to experimental condition and levels of immersion in the autobiographical memory task. In the present study, the primary focus was on cognitive outcomes, and this measure was not included as a theoretically driven predictor. Importantly, this questionnaire was administered prior to the encoding of the health messages and under identical conditions for all participants, reducing the likelihood that it differentially influenced experimental groups or the primary outcome of recall.*
