# Supplementary material for: Autobiographical memory of validating and invalidating consultations is associated with recall capacity for health information
Source: PLoS One. 2026 Jul 20;21(7):e0353615. doi: 10.1371/journal.pone.0353615 (PMC13384319; doi:10.1371/journal.pone.0353615)
Supplement: Peer review history

# Peer review: Decision Letter 0

**DOI:** 10.1371/journal.pone.0353615.r001

**Authors:** Igor Sotgiu

5 May 2026

PONE-D-26-06641

Autobiographical Memory of Validating and Invalidating Consultations is Associated with Recall Capacity for Health Information

PLOS One

Dear Dr. Lee,

Thank you for submitting your manuscript to PLOS ONE. After careful consideration, we feel that it has merit but does not fully meet PLOS ONE’s publication criteria as it currently stands. Therefore, we invite you to submit a revised version of the manuscript that addresses the points raised during the review process.

I was able to collect two reviews from two experts in your field. As you can see, both Reviewers raised a number of concerns that must be addressed before taking a final decision on the suitability of your article for publication. Overall, Reviewers’ comments concern methodological aspects of your study that were not appropriately described in the original version of your manuscript or that require the authors to provide relevant details that are currently missing (e.g., questions included the memory recall task, potential serial order effects, measures of inter-rater agreement, instructional manipulation attention checks, reasons for choosing the format of stimuli, additional questionnaire administered to participants, time interval between measurements). I agree with these comments and I encourage the authors to consider them in a revised version of their article to be completed over the next eight weeks. That said, I would like also to emphasize that while some of the Reviewers’ comments will necessarily determine changes in various sections of the article, other comments may be addressed in the point-by-point response letter.

Please submit your revised manuscript by Jun 19 2026 11:59PM. If you will need more time than this to complete your revisions, please reply to this message or contact the journal office at plosone@plos.org. When you're ready to submit your revision, log on to https://www.editorialmanager.com/pone/ and select the 'Submissions Needing Revision' folder to locate your manuscript file.

Please include the following items when submitting your revised manuscript:

- A letter that responds to each point raised by the academic editor and reviewer(s). You should upload this letter as a separate file labeled 'Response to Reviewers'.
- A marked-up copy of your manuscript that highlights changes made to the original version. You should upload this as a separate file labeled 'Revised Manuscript with Track Changes'.
- An unmarked version of your revised paper without tracked changes. You should upload this as a separate file labeled 'Manuscript'.

If you would like to make changes to your financial disclosure, please include your updated statement in your cover letter. Guidelines for resubmitting your figure files are available below the reviewer comments at the end of this letter.

If applicable, we recommend that you deposit your laboratory protocols in protocols.io to enhance the reproducibility of your results. Protocols.io assigns your protocol its own identifier (DOI) so that it can be cited independently in the future. For instructions see: https://journals.plos.org/plosone/s/submission-guidelines#loc-laboratory-protocols. Additionally, PLOS ONE offers an option for publishing peer-reviewed Lab Protocol articles, which describe protocols hosted on protocols.io. Read more information on sharing protocols at https://plos.org/protocols?utm_medium=editorial-email&utm_source=authorletters&utm_campaign=protocols.

As the corresponding author, your ORCID iD is verified in the submission system and will appear in the published article. PLOS supports the use of ORCID, and we encourage all coauthors to register for an ORCID iD and use it as well. Please encourage your coauthors to verify their ORCID iD within the submission system before final acceptance, as unverified ORCID iDs will not appear in the published article. *Only*  the individual author can complete the verification step; PLOS staff *cannot*  verify ORCID iDs on behalf of authors.

We look forward to receiving your revised manuscript.

Kind regards,

Igor Sotgiu, Ph.D.

Academic Editor

PLOS One

Journal Requirements:

When submitting your revision, we need you to address these additional requirements.

1. Please ensure that your manuscript meets PLOS ONE's style requirements, including those for file naming. The PLOS ONE style templates can be found at https://journals.plos.org/plosone/s/file?id=wjVg/PLOSOne_formatting_sample_main_body.pdf and https://journals.plos.org/plosone/s/file?id=ba62/PLOSOne_formatting_sample_title_authors_affiliations.pdf

2. We note that the grant information you provided in the ‘Funding Information’ and ‘Financial Disclosure’ sections do not match.

When you resubmit, please ensure that you provide the correct grant numbers for the awards you received for your study in the ‘Funding Information’ section.

3. Thank you for stating in your Funding Statement:

[This work supported by a joint and equal investment from UKRI [grant number MR/W004151/1] and the charity Arthritis UK [grant number 22891] through the Advanced Pain Discovery Platform (APDP) initiative. For UKRI, the initiative is led by the Medical Research Council (MRC), with support from the Biotechnology and Biological Sciences Research Council (BBSRC) and the Economic and Social Research Council (ESRC).

https://www.ukri.org/what-we-do/browse-our-areas-of-investment-and-support/advanced-pain-discovery-platform-apdp/

TP was a co-applicant on the grant supporting this work.

The funders did not play a role in the study design, data collection, analysis, decision to publish, or preparation of the manuscript.].

Please provide an amended statement that declares *all* the funding or sources of support (whether external or internal to your organization) received during this study, as detailed online in our guide for authors at http://journals.plos.org/plosone/s/submit-now.  Please also include the statement “There was no additional external funding received for this study.” in your updated Funding Statement.

Please include your amended Funding Statement within your cover letter. We will change the online submission form on your behalf.

4. Thank you for stating the following in the Competing Interests section:

[I have read the journal's policy and the authors of this manuscript have the following competing interests: TP provides consultation in psychologically informed practice, contracted to the University of Southampton.].

Please confirm that this does not alter your adherence to all PLOS ONE policies on sharing data and materials, by including the following statement: "This does not alter our adherence to PLOS ONE policies on sharing data and materials.” (as detailed online in our guide for authors http://journals.plos.org/plosone/s/competing-interests).  If there are restrictions on sharing of data and/or materials, please state these. Please note that we cannot proceed with consideration of your article until this information has been declared.

Please include your updated Competing Interests statement in your cover letter; we will change the online submission form on your behalf.

5. When completing the data availability statement of the submission form, you indicated that you will make your data available on acceptance. We strongly recommend all authors decide on a data sharing plan before acceptance, as the process can be lengthy and hold up publication timelines. Please note that, though access restrictions are acceptable now, your entire data will need to be made freely accessible if your manuscript is accepted for publication. This policy applies to all data except where public deposition would breach compliance with the protocol approved by your research ethics board. If you are unable to adhere to our open data policy, please kindly revise your statement to explain your reasoning and we will seek the editor's input on an exemption. Please be assured that, once you have provided your new statement, the assessment of your exemption will not hold up the peer review process.

6. We noted in your submission details that a portion of your manuscript may have been presented or published elsewhere.

[Aspects of this work have been presented at Pain Research Methods Scientific Meeting, 2024 and Advanced Pain Discovery Platform Scientific Meetings, 2024 & 2025.]

Please clarify whether this conference proceeding was peer-reviewed and formally published. If this work was previously peer-reviewed and published, in the cover letter please provide the reason that this work does not constitute dual publication and should be included in the current manuscript.

7. We note that there is identifying data in Table 1. Prior to sharing human research participant data, authors should consult with an ethics committee to ensure data are shared in accordance with participant consent and all applicable local laws.

Data sharing should never compromise participant privacy. It is therefore not appropriate to publicly share personally identifiable data on human research participants. The following are examples of data that should not be shared:

-Name, initials, physical address

-Ages more specific than whole numbers

-Internet protocol (IP) address

-Specific dates (birth dates, death dates, examination dates, etc.)

-Contact information such as phone number or email address

-Location data

-ID numbers that seem specific (long numbers, include initials, titled “Hospital ID”) rather than random (small numbers in numerical order)

Data that are not directly identifying may also be inappropriate to share, as in combination they can become identifying. For example, data collected from a small group of participants, vulnerable populations, or private groups should not be shared if they involve indirect identifiers (such as sex, ethnicity, location, etc.) that may risk the identification of study participants.

Additional guidance on preparing raw data for publication can be found in our Data Policy (https://journals.plos.org/plosone/s/data-availability#loc-human-research-participant-data-and-other-sensitive-data) and in the following article: http://www.bmj.com/content/340/bmj.c181.long.

Please remove or anonymize all personal information (sex, ethnicity, educational level, employment status), ensure that the data shared are in accordance with participant consent, and add an anonymized version to your manuscript file.

If the reviewer comments include a recommendation to cite specific previously published works, please review and evaluate these publications to determine whether they are relevant and should be cited. There is no requirement to cite these works unless the editor has indicated otherwise.

Please review your reference list to ensure that it is complete and correct. If you have cited papers that have been retracted, please include the rationale for doing so in the manuscript text, or remove these references and replace them with relevant current references. Any changes to the reference list should be mentioned in the rebuttal letter that accompanies your revised manuscript. If you need to cite a retracted article, indicate the article’s retracted status in the References list and also include a citation and full reference for the retraction notice.

[Note: HTML markup is below. Please do not edit.]

Reviewers' comments:

Reviewer's Responses to Questions

**Comments to the Author**

1. Is the manuscript technically sound, and do the data support the conclusions?

The manuscript must describe a technically sound piece of scientific research with data that supports the conclusions. Experiments must have been conducted rigorously, with appropriate controls, replication, and sample sizes. The conclusions must be drawn appropriately based on the data presented.

Reviewer #1: Yes

Reviewer #2: Yes

**********

2. Has the statistical analysis been performed appropriately and rigorously?

Reviewer #1: Yes

Reviewer #2: Yes

**********

3. Have the authors made all data underlying the findings in their manuscript fully available?

The PLOS Data policy requires authors to make all data underlying the findings described in their manuscript fully available without restriction, with rare exception (please refer to the Data Availability Statement in the manuscript PDF file). The data should be provided as part of the manuscript or its supporting information, or deposited to a public repository. For example, in addition to summary statistics, the data points behind means, medians and variance measures should be available. If there are restrictions on publicly sharing data—e.g. participant privacy or use of data from a third party—those must be specified.

Reviewer #1: Yes

Reviewer #2: Yes

**********

4. Is the manuscript presented in an intelligible fashion and written in standard English?

PLOS ONE does not copyedit accepted manuscripts, so the language in submitted articles must be clear, correct, and unambiguous. Any typographical or grammatical errors should be corrected at revision, so please note any specific errors here.

Reviewer #1: Yes

Reviewer #2: Yes

**********

5. Review Comments to the Author

Please use the space provided to explain your answers to the questions above. You may also include additional comments for the author, including concerns about dual publication, research ethics, or publication ethics. (Please upload your review as an attachment if it exceeds 20,000 characters)

Reviewer #1: Dear authors,

I enjoyed reading your work. Please find below some of my comments and suggestions for improving your manuscript.

1. In the abstract, I suggest you to include an opening sentence to contextualize the topic.

2. Line 58, the reference to (Timmerman et al 2016) has to be transformed into a number reference, in line with the others.

3. Line 70, I recommend clarifying the connection between what is presented in reference to the literature and the objectives of your study. Written in this way, it is unclear.

4. In general, in the method-participants section, I suggest you to include a specific note explaining in detail how the platform used (Prolific) works, so that even those unfamiliar with it can understand how participants are recruited, how they participate in the research, and how data are collected.

5. I suggest you to deeper detail the “payment information” (Line 103).

6. In the design and measures section, I suggest you to include the question asked to participants regarding autobiographical memory, so that the task of memory recall is clear to the reader as well. It is not clear if a questionnaire was used, or if there were questions with open response…please clarify this procedure better about the recall task.

7. Line 128, you write “To guide this task, examples of validating experiences included:”. Did you check that these examples included in the task did not influence the participants’ description of their autobiographical memories in any way? These examples could have acted as facilitators or suggestions, affecting memory’s dimensions (for example, the vividness of the memory, or the words used to describe the episode) and autobiographical recall. If this check was not carried out, you must include this as a possible limitation of the study.

8. Line 145, you write “These were then adapted to fit two categories:”. Please specify more clearly on what basis this choice was made, in terms of methodology.

9. Line 186, please specify what you mean by “Immersion in the autobiographical memory task”. Is this question intended to evaluate vividness, accuracy, emotional intensity, or something similar to these characteristics? And in which way can you argue that it is indeed a valid and reliable measure of your construct?

In this paragraph, you talk about “immersion” and then about “emotional engagement”. Please specify what do you mean.

10. Line 202-204 (and Line 215-216), I suggest you to remove “An additional 7-item questionnaire unrelated to the current analysis was also administered at this stage as part of a separate study.” because it is not in line with the study you are presenting in this manuscript.

11. When you analyzed the memory performance, did you take into account possible serial position effects, such as the primacy effect or the recency effect?

12. Line 263, please specify the “good power” reached by your research.

13. Please, italicize all letters referring to statistical indicators (e.g., alpha, etc.)

14. Please specify, the first time you use these letters, that V is for the validation group and I is for the invalidation group.

15. Are there differences in distribution by nationality within the groups and in the total sample? How is this monitored, if at all?

16. Table 3, p value column is not clear. Does it refer only to the invalidation group? Or to the comparison between the two groups? Do you have p value for the validation group?

17. Line 304, how was the “custom code written in R” created? What are the methodological foundations and bibliographical references used to create it?

18. Line 309, how many reviewers reviewed the 162 responses? And according to what procedure?

19. What type is the indicator of inter-judge agreement regarding the accuracy of revisions made to the random sample?

20. Line 344, missing an s --> vs

21. The conclusions summarize the results described above. I recommend including sections that focus more on the implications of the study and its connection with the clinical practice.

22. Line 398, I suggest inserting in the text the name of the author you are citing (ref. n. 16).

23. The autobiographical narratives produced by participants in the two groups could be significantly different in terms of length, vividness, quality, accuracy of the memory, the narrative coherence with which they are remembered and narrated, the emotional intensity of each event, the centrality of the specific event for the person, and its social sharing. Were these factors analyzed or taken into account in the analysis and presentation of the results? They may have played a role, even in the subsequent recall of health information.

24. Line 471, the closing round bracket is missing.

Reviewer #2: The manuscript addresses a relevant research question; however, several aspects require clarification and further development before the work can be fully evaluated. In particular, issues related to methodological transparency, justification of design choices, and interpretation of results should be addressed. Detailed comments are provided below:

1. The Introduction section would benefit from further expansion to more clearly situate the study within the existing literature and to better articulate the underlying rationale for the research.

2. In lines 80–81, the authors state that participants were required “to have experienced chronic pain for more than three months.” Please clarify how the presence and duration of chronic pain were verified, as this appears to rely on self-report.

3. In lines 90–91, the authors note that “instructional manipulation attention checks were embedded within the study” to ensure data quality. This aspect would benefit from a more detailed description, including examples of the attention checks used and how responses were handled.

4. In lines 95–96, the authors indicate that participants completed a retrospective self-report validity check (“Should we use your data?”). Please specify whether any participants responded negatively to this question and, if so, how these cases were treated in the analyses.

5. The manuscript does not indicate whether the study was pre-registered. The authors are encouraged to clarify this point and, if the study was not pre-registered, to provide a justification.

6. In lines 158–159, the authors state that “the 20 messages (10 action-oriented, 10 restriction-oriented) were delivered as audio recordings in a randomised order.” Please justify the decision to present the stimuli in audio format rather than in written form, and discuss any potential implications this choice may have for comprehension and recall.

7. In lines 202–204, the authors note that an additional 7-item questionnaire, unrelated to the current analysis, was administered as part of a separate study. It would be important to clarify how the authors ensured that these additional items did not interfere with participants’ responses in the present study. Furthermore, for transparency, the content of these items should be reported, either in the main text or as supplementary material.

8. In lines 209–210, the authors report that the “next” button appeared only after 3 minutes. However, earlier (lines 92–93), submissions completed “implausibly quickly” were excluded. The authors should clarify how submissions could be considered implausibly fast given this time restriction.

9. The manuscript would benefit from clarification regarding the time interval between T1 and T2. Please specify how much time elapsed between these two measurements.

10. In lines 252–253, the authors state that “15 descriptions were flagged as AI-generated (verified by Prolific) and were removed.” Additional detail is needed on how this verification process operates. Furthermore, please clarify how the authors can be confident that the remaining responses were not AI-generated.

11. In lines 312–313, the authors report that “a revised subset (10%) was then checked, and a high level of agreement (97.53%) was achieved.” It would be important to specify who conducted this agreement assessment, whether multiple raters were involved, and which metric of agreement was used.

12. The finding that validation moderated the change in fear from T1 to T2 warrants further explanation. The authors are encouraged to elaborate on the theoretical and/or empirical rationale underlying this effect.

13. Both Model fit indices indicate excellent fit; however, such perfect or near-perfect fit may raise concerns about model complexity or saturation. The authors should clarify the degrees of freedom and whether the model is just-identified.

**********

6. PLOS authors have the option to publish the peer review history of their article (what does this mean?). If published, this will include your full peer review and any attached files.

If you choose “no”, your identity will remain anonymous but your review may still be made public.

**Do you want your identity to be public for this peer review?**  For information about this choice, including consent withdrawal, please see our Privacy Policy.

Reviewer #1: No

Reviewer #2: **Yes:**  Federico Puleo

**********

[NOTE: If reviewer comments were submitted as an attachment file, they will be attached to this email and accessible via the submission site. Please log into your account, locate the manuscript record, and check for the action link "View Attachments". If this link does not appear, there are no attachment files.]

To ensure your figures meet our technical requirements, please review our figure guidelines: https://journals.plos.org/plosone/s/figures

You may also use PLOS’s free figure tool, NAAS, to help you prepare publication quality figures: https://journals.plos.org/plosone/s/figures#loc-tools-for-figure-preparation.

NAAS will assess whether your figures meet our technical requirements by comparing each figure against our figure specifications.

# Author comment: Author response to Decision Letter 1

**DOI:** 10.1371/journal.pone.0353615.r002

19 Jun 2026

We would like to thank both reviewers for their careful and constructive feedback on our manuscript. We have thoroughly considered all comments and have revised the manuscript accordingly. We believe that these revisions have strengthened the clarity, transparency, and overall quality of the manuscript. Below, we provide a point-by-point response to each comment and indicate where changes have been made in the revised manuscript.

Response to reviewer 1

1. In the abstract, I suggest you to include an opening sentence to contextualize the topic.

We thank the reviewer for this suggestion. We have now added a few sentences to the abstract to give more context (line 17).

2. Line 58, the reference to (Timmerman et al 2016) has to be transformed into a number reference, in line with the others.

Thank you for highlighting this, we have now corrected this reference in line with the journal style.

3. Line 70, I recommend clarifying the connection between what is presented in reference to the literature and the objectives of your study. Written in this way, it is unclear.

We thank the reviewer for this recommendation and have now expanded this section to explain the previous research and how the current study extends these findings in people living with chronic pain (lines 68-79)

4. In general, in the method-participants section, I suggest you to include a specific note explaining in detail how the platform used (Prolific) works, so that even those unfamiliar with it can understand how participants are recruited, how they participate in the research, and how data are collected.

We thank the reviewer for this suggestion and have added some more information on lines 88-93.

Participants were recruited through Prolific, an online research recruitment platform. Prolific maintains a participant pool in which users undergo verification procedures, including identity and location checks, as well as ongoing data quality monitoring [20]. Participants complete pre-screening questions and provide demographic information which researchers may use to advertise studies to eligible participants.

5. I suggest you to deeper detail the “payment information” (Line 103).

We have now added “the £3.50 reward for completion” for clarity (line122).

6. In the design and measures section, I suggest you to include the question asked to participants regarding autobiographical memory, so that the task of memory recall is clear to the reader as well. It is not clear if a questionnaire was used, or if there were questions with open response…please clarify this procedure better about the recall task.

We thank the reviewer for this helpful suggestion. We have revised the Design and Measures section to improve clarity regarding the autobiographical memory recall task. Specifically, we have added subheadings for the key tasks and included the full wording of the autobiographical memory instructions for each condition in a new table (Table 1). We have also added the exact instructions for the health information recall task in the Methods section (lines 183-192). These additions clarify that the autobiographical memory task involved open-ended recall rather than questionnaire-based responding.

7. Line 128, you write “To guide this task, examples of validating experiences included:”. Did you check that these examples included in the task did not influence the participants’ description of their autobiographical memories in any way? These examples could have acted as facilitators or suggestions, affecting memory’s dimensions (for example, the vividness of the memory, or the words used to describe the episode) and autobiographical recall. If this check was not carried out, you must include this as a possible limitation of the study.

We thank the reviewer for raising this important point. We acknowledge that the examples provided to illustrate validating and invalidating experiences may have influenced how participants constructed or worded their autobiographical memories. These examples were intended to standardise understanding of the manipulation and were closely matched across conditions to minimise systematic differences in interpretation.

We note that no group differences were observed in immersion ratings (Table 4), suggesting comparable levels of subjective reliving across conditions, although we acknowledge that this does not rule out potential influences of the instructional examples on the content or phrasing of autobiographical memory descriptions.

Importantly, the primary outcome of the present study was recall of separate health information, not the autobiographical memory narratives themselves. Any potential influence of the instructional examples on autobiographical memory descriptions would not be expected to differentially affect the between-group comparison of health information recall, as both groups received structurally equivalent instructions aside from the manipulation.

8. Line 145, you write “These were then adapted to fit two categories:”. Please specify more clearly on what basis this choice was made, in terms of methodology.

We thank the reviewer for this suggestion. We have expanded the explanation of this method of categorisation on lines 161-170. These categories were introduced as part of the message construction procedure based on observed differences in linguistic framing and functional intent of the health messages, and were used solely to ensure systematic development and a balanced stimulus set. They were not included as a factor in the primary analyses and served only to support careful control of message content during stimulus design.

9. Line 186, please specify what you mean by “Immersion in the autobiographical memory task”. Is this question intended to evaluate vividness, accuracy, emotional intensity, or something similar to these characteristics? And in which way can you argue that it is indeed a valid and reliable measure of your construct?

In this paragraph, you talk about “immersion” and then about “emotional engagement”. Please specify what do you mean.

We thank the reviewer for this helpful comment. Immersion in the autobiographical memory task was intended to capture the subjective experience of reliving the autobiographical memory, rather than objective accuracy, narrative detail, or vividness of recall. The wording of the item is provided in the Methods section (lines 129-121)

Although this is a single-item measure and has not been formally psychometrically validated, it was included as a theoretically grounded proxy for reliving during autobiographical recall, consistent with the aims of the study. We have added this clarification on line 221-223.

We agree that the use of “immersion” and “emotional engagement” required clarification. In the manuscript, both terms were intended to refer to the same construct, namely the subjective experience of reliving the autobiographical memory during the task. To improve clarity and consistency, we have standardised terminology throughout the manuscript to “immersion”.

10. Line 202-204 (and Line 215-216), I suggest you to remove “An additional 7-item questionnaire unrelated to the current analysis was also administered at this stage as part of a separate study.” because it is not in line with the study you are presenting in this manuscript.

We thank the reviewer for this suggestion and agree that the reference to the additional 7-item questionnaire is not necessary for the present manuscript give that it took place before the encoding of the recall messages. In line with this recommendation, we have removed this sentence from the Methods section.

11. When you analyzed the memory performance, did you take into account possible serial position effects, such as the primacy effect or the recency effect?

We thank the reviewer for raising this question. The same 20 health messages were presented to all participants. Serial position effects were addressed through randomisation of the presentation order of these messages for each participant. This is now clarified in the manuscript (lines 242-247).

Since items were randomised, any residual serial position effects would be expected to be evenly distributed across groups and are therefore unlikely to have biased the observed between-group comparisons. In addition, item-level differences affecting recall were accounted for in the statistical model by including random intercepts for health messages, ensuring that variability in message difficulty did not influence the estimated effects.

12. Line 263, please specify the “good power” reached by your research.

We have now clarified this on line 301.

13. Please, italicize all letters referring to statistical indicators (e.g., alpha, etc.)

We have now corrected italicisation of statistical indicators in the manuscript for consistency. In line with standard reporting conventions, Greek symbols (e.g., α) have been retained in their standard non-italicised form. However, we are happy to revise if PLoS ONE’s preferred style is for Greek letters to also be italicised.

14. Please specify, the first time you use these letters, that V is for the validation group and I is for the invalidation group.

We have now added this clarification on line 315.

15. Are there differences in distribution by nationality within the groups and in the total sample? How is this monitored, if at all?

We thank the reviewer for this question. Nationality was initially included in the descriptive characteristics of the sample; however, following further consideration and in line with journal data protection guidance, we removed the detailed breakdown due to small subgroup sizes and the risk of potential indirect identifiability when combined with other demographic variables (e.g., age, gender, and education). Aggregated demographic information is now reported in the manuscript in the text on lines 315-321 and in table 2.

Nationality was not included as a covariate in the primary analyses, as more theoretically relevant variables (pain intensity, pain duration, age, and sex) were prioritised. We acknowledge that cultural and national differences may nonetheless be relevant to experiences of validation and invalidation, and we have now noted this as an important direction for future research in the Discussion (line 541).

16. Table 3, p value column is not clear. Does it refer only to the invalidation group? Or to the comparison between the two groups? Do you have p value for the validation group?

Thanks to the reviewer for their attention to this aspect of the table. We have added a clarifying column heading to the table (now Table 4) and included a note specifying that independent-samples t-tests were conducted on participant-level change scores to compare differences in change between conditions. p values reflect between-group comparisons of change scores.

17. Line 304, how was the “custom code written in R” created? What are the methodological foundations and bibliographical references used to create it?

We thank the reviewer for this question and have clarified the development of this scoring code in the manuscript (lines 345-351).

18. Line 309, how many reviewers reviewed the 162 responses? And according to what procedure?

We thank the reviewer for this helpful question. We have revised the manuscript to clarify the scoring validation procedure (lines 351-360). Specifically, the automated scoring was manually validated by a single reviewer (the first author), who was blind to participant identity and condition.

To validate the scoring procedure, the first author manually scored a random 10 % sample of responses (n = 162), blind to participant identity and condition. These manual ratings were then compared against the automated scoring to assess agreement between human and algorithm-derived coding. Initial discrepancies were used to refine the keyword criteria in the code to better reflect the intended meaning of each message. A second subset (10 %) was then re-checked following revisions to the scoring algorithm and a high level of agreement (97.53 %) was achieved before full-dataset scoring. Agreement was calculated as simple percentage agreement between manual validation and automated scoring outputs.

19. What type is the indicator of inter-judge agreement regarding the accuracy of revisions made to the random sample?

The indicator reported (97.53%) reflects simple percentage agreement between the manually validated coding and the automated keyword-based scoring procedure. This represents the proportion of cases in which the automated output matched the manual validation within the randomly selected subset of responses. We have clarified this in the revised manuscript (lines 358-359).

20. Line 344, missing an s --> vs

This has now been corrected.

21. The conclusions summarize the results described above. I recommend including sections that focus more on the implications of the study and its connection with the clinical practice.

We thank the reviewer for this helpful suggestion. We have added a section in the Discussion about implications for clinical practice (lines 506-515) and revised the Conclusions section to place greater emphasis on the potential clinical implications of the findings (line 555 – 557). In particular, we now highlight the potential relevance of perceived clinician validation for patients’ recall of health information. We remain cautious about the limited clinical importance of these findings at this stage and emphasise the need for future research in this area.

22. Line 398, I suggest inserting in the text the name of the author you are citing (ref. n. 16).

We have now added the name of the author on line 436.

23. The autobiographical narratives produced by participants in the two groups could be significantly different in terms of length, vividness, quality, accuracy of the memory, the narrative coherence with which they are remembered and narrated, the emotional intensity of each event, the centrality of the specific event for the person, and its social sharing. Were these factors analyzed or taken into account in the analysis and presentation of the results? They may have played a role, even in the subsequent recall of health information.

We thank the reviewer for raising the important point that autobiographical narrative characteristics (e.g., length, vividness, emotional intensity, and coherence) may plausibly influence subsequent memory processes. We agree that these features may contribute to participants’ experiences of the autobiographical recall task, particularly as the narratives differed in content across experimental conditions (validation versus invalidation experiences).

However, the primary aim of the present study was to examine the effect of the experimental manipulation on subsequent outcomes, rather than to model the contribution of individual narrative characteristics. As such, detailed coding and analysis of narrative characteristics was not part of the present analyses and would constitute a separate level of analysis focused on language production rather than memory performance or task-level engagement. Additionally, characteristics such as narrative length or linguistic structure may reflect differences in recall style or expression that are not necessarily equivalent to engagement with the autobiographical experience.

Instead, participants’ self-reported immersion ratings were included as the primary index of engagement during the task, as this measure was designed to directly capture participants’ subjective experience of reliving the autobiographical memory and was considered the most theoretically aligned measure for the aims of this study.

We have clarified in the manuscript that the focus was on the subjective experience of validation/invalidation and its relationship with subsequent outcomes. In addition, we have added a section in the Discussion to address the limitation that autobiographical accounts reflect subjective and reconstructed memories rather than objectively verifiable events (lines 543-552)

24. Line 471, the closing round bracket is missing.

We have now corrected this (Line 539).

Response to Reviewer 2

1. The Introduction section would benefit from further expansion to more clearly situate

Attachment Submitted filename: Response to Reviewers.docx

# Editor report: Decision Letter 1

**DOI:** 10.1371/journal.pone.0353615.r003

**Authors:** Igor Sotgiu

23 Jun 2026

PONE-D-26-06641R1

Autobiographical Memory of Validating and Invalidating Consultations is Associated with Recall Capacity for Health Information

PLOS One

Dear Dr. Lee,

thank you for submitting your revised manuscript to PLOS ONE.

Overall, you and your co-authors did a very good job in revising your paper.

Indeed, I have just one comment on your revised article. In accordance with Reviewer 2 (and contrary to Reviewer 1), I invite you to report that an additional 7-item questionnaire was administered as part of a separate study. I agree with Reviewer 2 that this should be done for transparency. I also invite you to briefly describe the content of these items and the main reason why this instrument was not expected to influence your findings.

Please submit your revised manuscript by Aug 07 2026 11:59PM. If you will need more time than this to complete your revisions, please reply to this message or contact the journal office at plosone@plos.org. When you're ready to submit your revision, log on to https://www.editorialmanager.com/pone/ and select the 'Submissions Needing Revision' folder to locate your manuscript file.

Please include the following items when submitting your revised manuscript:

- A letter that responds to each point raised by the academic editor and reviewer(s). You should upload this letter as a separate file labeled 'Response to Reviewers'.
- A marked-up copy of your manuscript that highlights changes made to the original version. You should upload this as a separate file labeled 'Revised Manuscript with Track Changes'.
- An unmarked version of your revised paper without tracked changes. You should upload this as a separate file labeled 'Manuscript'.

If you would like to make changes to your financial disclosure, please include your updated statement in your cover letter. Guidelines for resubmitting your figure files are available below the reviewer comments at the end of this letter.

If applicable, we recommend that you deposit your laboratory protocols in protocols.io to enhance the reproducibility of your results. Protocols.io assigns your protocol its own identifier (DOI) so that it can be cited independently in the future. For instructions see: https://journals.plos.org/plosone/s/submission-guidelines#loc-laboratory-protocols. Additionally, PLOS ONE offers an option for publishing peer-reviewed Lab Protocol articles, which describe protocols hosted on protocols.io. Read more information on sharing protocols at https://plos.org/protocols?utm_medium=editorial-email&utm_source=authorletters&utm_campaign=protocols.

As the corresponding author, your ORCID iD is verified in the submission system and will appear in the published article. PLOS supports the use of ORCID, and we encourage all coauthors to register for an ORCID iD and use it as well. Please encourage your coauthors to verify their ORCID iD within the submission system before final acceptance, as unverified ORCID iDs will not appear in the published article. *Only*  the individual author can complete the verification step; PLOS staff *cannot*  verify ORCID iDs on behalf of authors.

We look forward to receiving your revised manuscript.

Kind regards,

Igor Sotgiu, Ph.D.

Academic Editor

PLOS One

Journal Requirements:

If the reviewer comments include a recommendation to cite specific previously published works, please review and evaluate these publications to determine whether they are relevant and should be cited. There is no requirement to cite these works unless the editor has indicated otherwise.

Please review your reference list to ensure that it is complete and correct. If you have cited papers that have been retracted, please include the rationale for doing so in the manuscript text, or remove these references and replace them with relevant current references. Any changes to the reference list should be mentioned in the rebuttal letter that accompanies your revised manuscript. If you need to cite a retracted article, indicate the article’s retracted status in the References list and also include a citation and full reference for the retraction notice.

[Note: HTML markup is below. Please do not edit.]

[NOTE: If reviewer comments were submitted as an attachment file, they will be attached to this email and accessible via the submission site. Please log into your account, locate the manuscript record, and check for the action link "View Attachments". If this link does not appear, there are no attachment files.]

To ensure your figures meet our technical requirements, please review our figure guidelines: https://journals.plos.org/plosone/s/figures

You may also use PLOS’s free figure tool, NAAS, to help you prepare publication quality figures: https://journals.plos.org/plosone/s/figures#loc-tools-for-figure-preparation.

NAAS will assess whether your figures meet our technical requirements by comparing each figure against our figure specifications.

# Author comment: Author response to Decision Letter 2

**DOI:** 10.1371/journal.pone.0353615.r004

24 Jun 2026

In accordance with Reviewer 2 (and contrary to Reviewer 1), I invite you to report that an additional 7-item questionnaire was administered as part of a separate study. I agree with Reviewer 2 that this should be done for transparency. I also invite you to briefly describe the content of these items and the main reason why this instrument was not expected to influence your findings.

Thank you for reviewing our revised manuscript, and for your positive comments about the changes made. We agree that transparency is important, and are happy to include more details about the additional questionnaire that formed part of a separate study.

Procedural information was added on lines 234-238 and 250-251:

A momentary body awareness questionnaire [32] was administered at this stage as part of a separate study not related to the present research question. This measure assessed self-reported awareness of bodily sensations across seven domains (heartbeat, breathing, posture, skin, muscles, stomach/intestines, and joints) on a 0 (not at all) to 10 (very much) scale.

Participants answered follow-up questions about the healthcare provider and setting they had described and completed the same body awareness questionnaire again (part of the separate study as described above).

We have also added a discussion of this as a potential limitation on lines 559-569:

A final limitation to consider is the inclusion of a brief momentary body awareness questionnaire as part of a separate study. Although this measure was not related to the primary cognitive outcomes of this study, it was completed within the same experimental session. Exploratory analyses of this measure, reported in a separate manuscript currently under review, suggest that body awareness was relatively stable over time, with small variations related to experimental condition and levels of immersion in the autobiographical memory task. In the present study, the primary focus was on cognitive outcomes, and this measure was not included as a theoretically driven predictor. Importantly, this questionnaire was administered prior to the encoding of the health messages and under identical conditions for all participants, reducing the likelihood that it differentially influenced experimental groups or the primary outcome of recall.

Attachment Submitted filename: Response_to_reviewers_auresp_2.docx

# Editor report: Decision Letter 2

**DOI:** 10.1371/journal.pone.0353615.r005

**Authors:** Igor Sotgiu

25 Jun 2026

Autobiographical Memory of Validating and Invalidating Consultations is Associated with Recall Capacity for Health Information

PONE-D-26-06641R2

Dear Dr. Lee,

We’re pleased to inform you that your manuscript has been judged scientifically suitable for publication and will be formally accepted for publication once it meets all outstanding technical requirements.

Within one week, you’ll receive an e-mail detailing the required amendments. When these have been addressed, you’ll receive a formal acceptance letter and your manuscript will be scheduled for publication.

An invoice will be generated when your article is formally accepted. Please note, if your institution has a publishing partnership with PLOS and your article meets the relevant criteria, all or part of your publication costs will be covered. Please make sure your user information is up-to-date by logging into Editorial Manager at Editorial Manager® and clicking the ‘Update My Information' link at the top of the page. For questions related to billing, please contact billing support.

If your institution or institutions have a press office, please notify them about your upcoming paper to help maximize its impact. If they’ll be preparing press materials, please inform our press team as soon as possible -- no later than 48 hours after receiving the formal acceptance. Your manuscript will remain under strict press embargo until 2 pm Eastern Time on the date of publication. For more information, please contact onepress@plos.org.

Kind regards,

Igor Sotgiu, Ph.D.

Academic Editor

PLOS One

Additional Editor Comments (optional):

Reviewers' comments:

# Editor report: Acceptance letter

**DOI:** 10.1371/journal.pone.0353615.r006

**Authors:** Igor Sotgiu

PONE-D-26-06641R2

PLOS One

Dear Dr. Lee,

I'm pleased to inform you that your manuscript has been deemed suitable for publication in PLOS One. Congratulations! Your manuscript is now being handed over to our production team.

At this stage, our production department will prepare your paper for publication. This includes ensuring the following:

* All references, tables, and figures are properly cited

* All relevant supporting information is included in the manuscript submission,

* There are no issues that prevent the paper from being properly typeset

You will receive further instructions from the production team, including instructions on how to review your proof when it is ready. Please keep in mind that we are working through a large volume of accepted articles, so please give us a few days to review your paper and let you know the next and final steps.

Lastly, if your institution or institutions have a press office, please let them know about your upcoming paper now to help maximize its impact. If they'll be preparing press materials, please inform our press team within the next 48 hours. Your manuscript will remain under strict press embargo until 2 pm Eastern Time on the date of publication. For more information, please contact onepress@plos.org.

You will receive an invoice from PLOS for your publication fee after your manuscript has reached the completed accept phase. If you receive an email requesting payment before acceptance or for any other service, this may be a phishing scheme. Learn how to identify phishing emails and protect your accounts at https://explore.plos.org/phishing.

If we can help with anything else, please email us at customercare@plos.org.

Thank you for submitting your work to PLOS ONE and supporting open access.

Kind regards,

PLOS ONE Editorial Office Staff

on behalf of

Prof. Igor Sotgiu

Academic Editor

PLOS One
